# "How about me giving blood for the COVID vaccine and not being able to get vaccinated?" A cognitive interview study on understanding of and agreement with broad consent for future use of data and samples in Colombia and Nicaragua

**DOI:** 10.1371/journal.pgph.0001253

**Published:** 2023-05-17

**Authors:** Lauren Maxwell, Jackeline Bravo Chamorro, Luz Marina Leegstra, Harold Suazo Laguna, María Consuelo Miranda Montoya

**Affiliations:** 1 Heidelberger Institut für Global Health, Universitätsklinikum Heidelberg, Heidelberg, Germany; 2 Facultad de Salud, Universidad Industrial de Santander, Bucaramanga, Santander, Colombia; 3 Sustainable Sciences Institute, Managua, Nicaragua; Public Health Foundation of India, INDIA

## Abstract

Broad consent for future use, wherein researchers ask participants for permission to share participant-level data and samples collected within the study for purposes loosely related to the study objectives, is central to enabling ethical data and sample reuse. Ensuring that participants understand broad consent-related language is key to maintaining trust in the study and public health research. We conducted 52 cognitive interviews to explore cohort research participants’ and their parents’ understanding of the broad consent-related language in the University of California at Berkeley template informed consent (IC) form for biomedical research. Participants and their parents were recruited from long-standing infectious disease cohort studies in Nicaragua and Colombia and interviewed during the COVID-19 pandemic. We conducted semi-structured interviews to assess participants’ agreement with the key concepts in the IC after clarifying them through the cognitive interview. Participants did not understand abstract concepts, including collecting and reusing genetic data. Participants wanted to learn about incidental findings, future users and uses. Trust in the research team and the belief that sharing could lead to new vaccines or treatments were critical to participant support for data and sample sharing. Participants highlighted the importance of data and sample sharing for COVID-19 response and equitable access to vaccines and treatments developed through sharing. Our findings on participants’ understanding of broad consent and preferences for data and sample sharing can help inform researchers and ethics review committees working to enable ethical and equitable data and sample sharing.

## Introduction

Research funders, regulatory agencies, and journals increasingly advocate for or require that individual-level health research data be shared to maximize utility [1, 2]. The advantages of sharing data and samples include gaining new insights, improving research transparency, and reducing the burden and costs of unnecessary duplication of research, which is especially important for low-and-middle-income countries (LMICs), which face the competing challenges of the highest burden of infectious disease (ID) and limited resources for research and biobanking [3–5].

Informed consent (IC) ensures participants’ voluntary participation in research and their understanding of the direct risks and benefits associated with participation [6]. IC is critical to the ethical conduct of research (principle of respect for persons) and is vital for enrolment and continuity of involvement in cohort studies [3, 7]. A commonly used and widely accepted measure of consent for data sharing is broad consent, wherein consent is obtained simultaneously for the primary study and for future use of the data and samples for a loosely specified range of future research purposes that go beyond the objectives of the original study [8]. The wording used for broad consent may explicitly state or imply that participants will not be re-contacted when sharing their data [9]. The consent form may or may not describe the criteria for releasing data for these future uses and which group or groups will make those decisions, i.e., the data governance framework for future use.

Understanding and addressing ethical and cultural concerns regarding data and sample sharing is central to developing values-based, ethical, and appropriate IC practices. Understanding how to explain data sharing to research participants was cited as a priority for future research in a recent systematic review of barriers to data sharing in LMICs and in primary research with research participants, ERC members, and researchers [3, 5, 10]. While several qualitative studies in LMICs have been conducted on the importance of consent for data sharing and participants’ preferred methods for obtaining consent [3, 5, 7, 11, 12], very few studies have assessed participants’ understanding of broad consent [4, 9]. Broad consent for data and sample sharing adds a layer of complexity to the consent process as it involves explaining abstract concepts to research participants [4]. Participants’ understanding of future use and incidental findings is crucial for cohort studies and perhaps even more critical for ID-focused cohort studies in LMICs, whose longevity is dependent on the continued trust of the community and the local and national public health authorities who are responsible for moderating communication around IDs and seen as ultimately responsible for sharing benefits derived from data collected from their constituents.

### Research objective

This project explored research participants’ and their parents or legal guardian’s understanding of the Spanish-language translation of the University of California at Berkeley (UCB) template IC for biomedical research that includes broad consent for clinical-epidemiological and genetic data and sample sharing and the Moore Clause [13]. The Moore Clause, which informs research participants that they will not have the right to claim compensation for commercial products or other intellectual property developed using their biospecimens from human subjects research [14], is related to a lawsuit brought by a California research participant whose biopsy tissue was used without his knowledge for commercial purposes. We included participants from a dengue cohort site in Nicaragua, which has a long-standing collaboration with UCB researchers, and from a dengue cohort in Colombia to compare the understanding of and attitudes toward the template language across the two contexts.

## Materials and methods

Cognitive interviewing is a qualitative research method that explores how research participants understand specific language (e.g., survey questions, survey directions) and how that understanding varies across contexts [15]. We conducted cognitive interviews to explore research participants’ and their parents’ understanding of the broad consent-related language in the UCB IC form. We provide the text from the UCB template IC in S1 Table. After working to ensure participants understood the critical concepts in the broad consent, we used a semi-structured interview to ascertain their agreement with those concepts and a demographic questionnaire to identify factors associated with understanding of or agreement with data and sample sharing (age group, gender, education level, occupation).

We began with four pilot interviews with the UCB template in Colombia and three in Nicaragua to refine the cognitive interview format and semi-structured interview guide. Following the pilot, we conducted the first round of cognitive interviews with the UCB template, with 20 participants in Colombia and 17 in Nicaragua. We then substituted the UCB template consent language with terms provided by cohort participants and their parents. We evaluated understanding of the revised IC with four new participants at each site.

The study protocol and interview guides are available in Spanish and English on the OSF website (DOI 10.17605/OSF.IO/UXVHK). This study is reported in keeping with the Cognitive Interview Reporting Framework (CIRF) Guidelines [16], with the exclusion of guidelines specific to evaluating questionnaires that were not relevant to this study. Results from the thematic analysis are presented in keeping with the Standards for Reporting Qualitative Research [17]. The Spanish language version of the UCB template IC, IDI guide, and the thinking-aloud exercise are included in S1 Text. The community-informed broad consent language is provided in S2 Text.

### Study population & recruitment

Participants were adults (aged 18 and over) who could provide IC and were the parent or guardian of a child who participated in an ongoing community- or hospital-based dengue virus-related cohort study (N = 42) or, in the case of Colombia, themselves cohort study participants (N = 10). In Nicaragua, we could not enroll cohort participants because they were younger at enrolment into the source cohort study, and the source cohort was launched later than in Colombia. Hence, cohort participants were younger than 18 at the time of this study. In Nicaragua, we excluded parents or guardians of participants younger than six months old or whose child weighed less than eight kilos at the time of enrolment and participants that lived outside the State of Managua from the study because these participants had higher levels of follow-up than the general cohort or could not be reached for the in-person interview, respectively.

In Colombia, we recruited participants from the list of parents and participants in the cohort study "Identification of priority age groups to be vaccinated against dengue in children and adolescents," which began in 2012 and included participants aged 16–25. In Nicaragua, we recruited participants from a current list of parents of participants in the "Prospective hospital-based study of dengue classification, case management, and diagnosis in Nicaragua" study, assembled between 2015–2018. Inclusion criteria for the Colombia [18] and Nicaragua [19] source cohort studies are described elsewhere. To capture the diversity in how cohort participants perceive broad consent language in the IC, we grouped each country-specific randomly ordered list of participants by education level (completed versus did not complete primary school) and gender. Participants were then selected from one of the four groupings at each site (e.g., male, completed primary school) by going sequentially through the previously randomized grouping and selecting one participant from each group for that round. Few men or participants with lower than primary education were included in the lists, so participants were not evenly distributed across these four groups.

Given the ongoing nature of the source cohort studies, cohort staff communicated regularly with participants. A field interviewer from each study approached potential participants during a regularly scheduled study visit or follow-up call and described the project using the recruitment script. Participants that consented to learn more about the cognitive interview study were then contacted by a study team member. The interviews occurred between August 2021 and April 2022 during the COVID-19 pandemic. Contacts took place over the phone in Colombia and in person in Nicaragua in keeping with local directives around maintaining distance and ventilation. Because the assessment of participant understanding of IC was not considered sensitive, we secured verbal IC in Colombia and written IC in Nicaragua. When parents or participants refused participation, we moved to the next parent, legal guardian, or research participant on the list for that group (e.g., completed primary education, female).

### Cognitive interview

We used verbal probing and think-aloud methods in the cognitive interviews [16, 20]. Verbal probing was used to clarify participants’ understanding of complex concepts, including genetic data, de-identified data, and data and sample sharing. The think-aloud method was used to understand the respondents’ thought or decision-making process and how or why participants decided to accept or reject broad consent for future use. The classical approach to cognitive interviewing, developed by Tourangeau [21], includes how participants (1) understand the question and answers, (2) retrieve information (recall), (3) weigh different answers (estimation or judgment), and (4) arrive at their decision (response). Because we were using text from the informed consent rather than a questionnaire, participants’ recall and response processes were irrelevant to our analysis and we used a modified version of the Tourangeau model limited to comprehension and judgment.

Before beginning the pilot interviews, interviewers met for several weeks to practice the pre-interview "think aloud" exercise and refine the interview guide. We evaluated how participants understood certain words and phrases and entire interview sections. We used probes like "tell me more about that" to explore different interpretations of the IC. After participants explained what came to mind, we used their language to describe what we saw as the intent behind the related text (e.g., data sharing, genetic data, incidental findings) and asked participants to then describe what they then understood. We iterated this process until the participant could explain in their own words what we understood as the intended meaning of the broad consent-related language. We conducted the cognitive and semi-structured interviews in Spanish. In Nicaragua, interviews were conducted face-to-face and recorded on a digital recorder. In Colombia, interviews were conducted and recorded by smartphone.

#### Pilot phase

The study IC, cognitive interview guide, warm-up exercises, and semi-structured interview guide were pilot tested with participants or their guardians from each cohort to ensure the cultural appropriateness of our study’s IC and interview guide language. After the pilot, we shortened the cognitive interview and divided the consent and the introduction to the "think aloud" exercise and the interview itself into two separate visits to shorten the interview duration and give participants additional time to process. We also switched the focus of the mock study presented in the cognitive interview from dengue to Zika virus to prevent confusion, as participants initially consented to their child’s participation or assented to their own participation in a dengue-focused cohort study.

#### Cognitive interviews with UCB template and community-informed language

Following the cognitive interviews conducted with the UCB template IC between March 2021 and February 2022, we used participants’ responses to revise the broad consent language in the template IC. We chose the revised language from participants’ own descriptions of the complex concepts in the IC and through discussions between representatives of the cohort source community and the analysis team. We then evaluated the understanding of the community-informed IC with four additional participants at each site between March and April 2022.

### Analysis

Interview recordings were transcribed verbatim, and the de-identified transcripts were uploaded to MAXQDA [22] for analysis by the study team who are fluent in Spanish. Interviews were conducted, transcribed, and analyzed on a rolling basis to allow us to identify emerging interpretations and themes. At least two team members independently reviewed all transcripts, and all five team members reviewed and evaluated a subset of transcripts.

We used thematic analysis based on Colaizzi’s model of descriptive phenomenology [23, 24] to explore participants’ attitudes toward data and sample sharing. During the first phase of the analysis, we developed preliminary deductive codes and definitions based on (1) the interview guide; (2) literature identified through a systematic search [25]; and (3) our own experience as researchers in public health research-related data and sample sharing. We developed inductive codes by reviewing interviewer field notes, written transcripts, and analytic memos. In the initial stage, the team read transcripts several times so that we could familiarise ourselves with the data and jointly develop the codebook. Following the joint analysis stage, to ensure reliability, JBC and LML met weekly to resolve differences in the interpretation of cognitive interview findings and the application of codes and identification of emergent inductive codes grounded in the transcript data. During the same period, the entire team held weekly discussions to update and refine the codebook, clarify local expressions with the help of the field interviewers (JBC, HSL), and discuss emerging themes. During the second phase of the analysis, the team met weekly to group the interpretations of the text and codes into meaningful themes and discuss whether themes varied across research site, gender, level of education, and cohort participants or their parents and guardians. We resolved differences of opinion in interpreting the cognitive interview and thematic analysis findings through consensus. Codes are presented in S2 Table.

#### Saturation

We defined saturation [26] in relation to both the cognitive interview and the thematic analysis of participant attitudes towards data and sample sharing. We stopped conducting interviews after we did not identify (1) new ways that participants understood broad consent for future use language or (2) new themes in respondents’ attitudes towards broad consent. Saturation was considered both within and between sites and other subgroupings, including gender and cohort participant versus parent or legal guardian.

#### Reflexivity

Participants in Colombia were interviewed by a Colombian nurse and bioethicist (JBC). In Nicaragua, participants were interviewed by an economist and expert in community-based participatory research (CBPR; HSL). Additional team members come from the US (LM), Argentina (LML), and Colombia (MCMM). All team members have or currently work in Latin America. Team members are engaged in data and sample sharing projects and have experience conducting and analyzing qualitative research, including cognitive interviews and CBPR. All team members reviewed translations for fidelity to local variations in Spanish. We provide the original Spanish-language version of all English-language translations of quotes included in this manuscript in S3 Table.

### Ethics statement

The study protocol and forms were approved by the Centro de Atención y Diagnóstico de Enfermedades Infecciosas (INFOVIDA; Bucaramanga, Colombia), Instituto de Ciencias Sostenibles (Managua, Nicaragua), and Universitätsklinikum Heidelberg (Heidelberg, Germany) ERCs. Further ethics considerations are reported in the PLOS Inclusivity in Global Research Questionnaire (S3 Text).

## Results

Table 1 summarises basic demographic information for study participants across sites. At both sites, we asked women if we could talk to their male partners about their child’s participation in the study because very few men were registered as parents or legal guardians in the list of cohort participants. The fathers we spoke to reported that they were unaware of their child’s participation in the original cohort study that was the source of participants for this study.

**Table 1:** Participant characteristics (N = 52) by site and whether they were the parents or tutors of cohort participants or themselves participants in the source cohort.

	Colombia (N = 28)				Nicaragua (N = 24)				Total (N = 52)			
	Parent or tutor		Cohort participant		Parent or tutor*		Cohort participant		Parent or tutor		Cohort participant	
Broad consent language	N	(%)	N	(%)	N	(%)	N	(%)	N	(%)	N	(%)
**Pilot with UCB IC**	3	(17)	1	(10)	3	(13)	0	(0)	6	(14)	1	(10)
**UCB IC**	13	(72)	7	(70)	17	(71)	0	(0)	30	(71)	7	(70)
**Community-informed IC**	2	(2)	2	(20)	4	(17)	0	(0)	6	(14)	2	(20)
**Age**												
**under 25**	0	(0)	7	(70)	0	(0)	0	(0)	0	(0)	7	(70)
**25–34**	2	(11)	2	(20)	3	(13)	0	(0)	5	(12)	2	(20)
**35–45**	8	(44)	1	(10)	14	(58)	0	(0)	22	(52)	1	(10)
**over 45**	8	(44)	0	(0)	7	(29)	0	(0)	15	(36)	0	(0)
**Gender**												
**Male**	2	(11)	5	(50)	4	(17)	0	(0)	6	(14)	5	(50)
**Female**	16	(89)	5	(50)	20	(83)	0	(0)	36	(86)	5	(50)
**Other**	0	(0)	0	(0)	0	(0)	0	(0)	0	(0)	0	(0)
**Education**												
**No formal education**	0	(0)	0	(0)	1	(4)	0	(0)	1	(2)	0	(0)
**Some primary school**	1	(6)	0	(0)	2	(8)	0	(0)	3	(7)	0	(0)
**Completed primary school**	3	(17)	0	(0)	0	(0)	0	(0)	3	(7)	0	(0)
**Some high school**	4	(22)	0	(0)	7	(29)	0	(0)	11	(26)	0	(0)
**Completed high school**	5	(28)	3	(30)	8	(33)	0	(0)	13	(31)	3	(30)
**Any post-secondary education**	5	(28)	7	(70)	6	(25)	0	(0)	11	(26)	7	(70)
**Employment**												
**Work inside home**	4	(22)	1	(10)	10	(42)	0	(0)	14	(33)	1	(10)
**Work outside the home**	13	(72)	7	(79)	13	(54)	0	(0)	26	(62)	7	(70)
**Student**	0	(0)	2	(20)	0	(0)	0	(0)	0	(0)	2	(20)
**Unemployed**	1	(6)	0	(0)	1	(4)	0	(0)	2	(5)	0	(0)

IC = informed consent, UCB = University of California at Berkeley

*Includes 4 tutors

Out of 52 cognitive interviews, 11 (21%) were conducted with men. No men participated in the pilot, but three men participated in interviews with the community-informed consent. Most participants were between 35–45 years old (N = 23; 44%) or over 45 (N = 15; 29%). Younger participants were more likely to have completed post-secondary education and to work outside the home than older participants. Colombia, which enrolled cohort participants (N = 10), had a higher proportion of younger participants and participants with higher levels of education than Nicaragua. One cohort participant completed the pilot interview, and two participated in the cognitive interview with the community-informed consent. Most parents or tutors of cohort participants worked outside the home (N = 26; 62%) rather than within the home (N = 14; 33%). While 15 of the 41 female participants (37%) reported working within the home, no male participants (N = 11) reported working there. Challenges in understanding keywords, phrases, and concepts in the broad consent section of the IC form were relatively consistent across participants in Nicaragua and Colombia. Variation in how participants understood the broad consent language in the UCB template IC is presented in Table 2 and described in detail below.

**Table 2:** Understanding of key concepts in the University of California Berkeley informed consent template (N = 44*) and community-informed revised informed consent (N = 8).

Concept	Text from UCB IC	Interpretation	N interpretation (N = 44)	Revised text	Interpretation	N interpretation (N = 8)
Storing & sharing genetic data	We would like to have additional permission to store your child’s clinical information, blood samples and DNA for future genetic studies. Genetic studies examine the genetic information (represented by DNA) passed on from parents to children.	No idea what genetic studies are	24	We would like to have additional permission to store your child’s clinical information, blood samples and DNA for future genetic studies. Genetic studies examine the genetic information (represented by DNA) passed on from parents to children. Each person has his or her own physical characteristics and even his or her way of being thanks to the genes that are passed on from parents to children. These genes are organised in living beings like parts of a puzzle and the one in charge of putting the puzzle together is the DNA. A fully assembled puzzle is called a genome. Sometimes the DNA has faults, it does not do its job well and the puzzle is not well assembled. When the puzzle is not well assembled, certain diseases appear in people. That is why scientists are dedicated to analysing the DNA, to check which genes are not well organised and determine whether they can develop specific drugs for helping correct that disease.	No idea what genetic studies are	1
		Genetic studies refer to paternity tests or descendants	7		Genetic studies refer to paternity tests or descendants	2
		Blood type	5		Blood type	1
		Genetic studies are related to other experiments that are not public health-related	1		Genetic studies are related to other experiments that are not public health-related	0
		Genetic manipulation/cloning	1		Genetic manipulation/cloning	0
		Genetic studies relate to the study of diseases that are transmitted from parents to children	6		Genetic studies relate to the study of diseases of diseases that are transmitted from parents to children	0
		Concept understood	0		Concept understood	4
Sample	Biological samples	Samples are blood or urine that are stored for research	18	Blood sample		0
		Samples are blood that are used for transfusion	1			0
		Concept of biological samples not understood, cannot put in their own words	11			0
		Concept understood	14		Concept understood with an additional explanation provided	8

IC = informed consent, UCB = University of California at Berkeley

*Includes 7 pilot interviews conducted with the UCB template IC

### Genetic data

The terms "genetic studies," "genetic data," "DNA," and "RNA" were not well understood. Participants described "genetic studies" as cloning or paternity detection when asked to put these terms in their own words. Thirty-one of the 44 participants across the two sites had trouble explaining what a genetic study referred to.

*"Genetic studies*? *Are they going to look for genes from other people in my son’s blood*? *That’s more or less what I understand*.*"*

(P6-ASU, Colombia, 35–45 years old, female, respondent’s parent)

*"Genetic studies are the samples they ask for the parents to know if the child is theirs or not*.*"*

(N20-MSMR, Nicaragua, over 45 years old, female, respondent’s parent)

Participants generally understood the reference to commercial products in the IC and that the research results could become the basis for new commercial products but expressed concerns about benefit sharing, including access to medications and vaccines developed through data and sample reuse. The phrase "diagnostic tests or therapeutic agents" was not well understood.

*"Well*, *the diagnostic tests are those of the laboratory*, *right*? *I don’t understand the other*.*"*

(P6 ASU, Colombia, 35–45 years old, female, respondent’s parent)

### Sample storage

The concept of storing biospecimens was generally well understood and accepted by participants. The term "biobank" was associated with "a refrigerator" and a "blood bank" to preserve and safeguard the samples—participants associated biobanking with "preserving the blood" for future research.

*"*…*just like a blood bank*, *they should keep them in a warehouse*, *in a totally cold room*…*so that the samples are not damaged*.*"*

(C22-DAR, Colombia, less than 25 years old, male, participant)

In a few cases, participants took storage of blood samples to mean blood donation as for transfusions.

*"*…*it is where they store blood samples or blood types when people need them*, *and I think that is what donations are for*, *and they receive them in case of an emergency"*.

(N6-MPH, Nicaragua, 35–45 years old, female, respondent’s parent)

*"Let’s say one bad thing is in the case that*, *let’s say*, *she suffers from a chronic disease and you come and*, *let’s say*, *the institution came*, *and they took the sample from my daughter and the person who received that blood*, *got sick*.*"*

(N19 JALC, Nicaragua, 35–45 years old, male, respondent’s parent)

### Confidentiality

Most participants understood the confidentiality-related procedures described in the consent form, including removing direct identifiers (e.g., name, address) and assigning a code to data and samples. Participants expressed concern that the steps taken to protect participant confidentiality could mean that clinically relevant results could not be communicated to research participants.

*"I am worried because this tells me that all data will be erased*. *To a certain extent*, *I do not agree with that idea because it makes sense that there won’t be any negative consequences*. *But at least I say that if you get hit [you were infected]*, *you should be informed*.*"*

(N17-JGG, Nicaragua, over 45 years old, male, respondent’s parent)

In contrast, one participant expressed the concern that sharing detailed results from genetic research could lead to the identification of individual participants.

*"Since it’s for future Zika research*, *the concern was that they would find something and say*, *’In such-and-such a person*, *we found something in their genes*!*’ Or what do I know*, *some kind of disease and publish it*, *and the person’s name would be revealed*.*"*

(N12- MMAG, Nicaragua, 35–45 years old, female, respondent’s parent)

Another participant expressed concern that the steps taken to ensure confidentiality might not be sufficient.

*"In some cases*, *I feel good*, *and in other cases*, *not because there is always the idea*: *what will happen to the sample*? *And if the information is really going to be confidential*, *what one fears the most as a parent is that something is going to happen to the child because we are giving so much information about him*.*"*

(N5-CMAZ, Nicaragua, 35–45 years old, female, respondent’s parent)

### Incidental findings

The UCB template allows research teams to choose whether or not to disclose incidental findings from research-related genetic testing but recommends that researchers not disclose these findings to research participants [13]. As such, we chose the option to not disclose incidental findings in the template version used in this study. The phrase "clinically relevant results" was well understood and associated with a diagnosis or a critical health condition.

*"Clinically relevant is when the results are bad*. *When something unexpected comes out*, *that would be relevant to the child*.*"*

(P13-LDB, Colombia, 25–34 years old, female, respondent’s parent)

Research participants generally did not understand that they would not be told about incidental findings from future studies that reused their data or samples. Participants expected that these findings would be shared and disagreed with the language in the template IC once it was clarified.

*"I see it as bad that they keep that result if in any case*, *there is some type of disease that they discover*, *apart from what we are doing*, *because the blood sample study is not only for Zika*. *You are telling me that there will be other types of exams or studies that are going to be done on the samples*, *then if you tell me that there is not going to be any individual result or that they are going to tell the person I don’t think it’s right*, *in that sense I put the case when a person is a blood donor*.*"*

(N17 JGG, Nicaragua, over 45 years old, male, respondent’s parent)

*"I think we have the right to know if something suddenly came out of that study that could affect my son’s health*.*"*

(P13-LDB, Colombia, 25–34 years old, female, respondent’s parent)

*"*…*if something relevant comes out*, *then yes*, *I would like them to tell me*.*"*

(P4-JS, Colombia, over 45 years old, female, respondent’s parent)

*"I am aware that you are going to carry out the study*. *Perhaps that way they will produce a vaccine to cure Zika*. *But if the child has something relevant*, *you will not tell me*, *and that worries me*.*"*

(NPILOTO2-ESLO, Nicaragua, over 45 years old, female, respondent’s parent)

*"My way of thinking was that if something abnormal happened in her blood type*, *it would be communicated*, *but you tell me that it would not be revealed*. *Practically*, *[the information] is for you*, *and that’s it*. *Is that right*?*"*

(N3-CCBB, Nicaragua, 35–45 years old, female, respondent’s parent)

*"That they are going to share my blood*, *and well*, *in actuality*, *I would think that would be wrong because you have to know*….*It could be that they do something wrong or that there is some illness and you don’t know about it*, *and afterward*, *you are sick and*, *and you don’t even find out*. *That’s why it would be bad*.*"*

(P11-EYP, Colombia, 35–45 years old, female, respondent’s parent)

### Broad consent for future use

Participants understood that pseudonymized data and samples could be shared in the future with researchers that study other diseases or viruses and generally agreed with this concept. In one case, broad consent was interpreted as a way for investigators to "clean their hands" of responsibility to the research participants because, when identifying information is removed, investigators can reuse the data or sample without needing to inform the participant of the results or concerning themselves with protecting the participants’ confidentiality.

*"Well*, *I already authorized them to use it for the Zika study*, *and I also authorized the deletion of the data once*, *so*, *well*, *I authorized it*, *right*? *Because it is like a form that is being filled*, *right*? *To be able to rotate it*, *to be able to give it to other researchers*, *then it is as if it is already there as if they are cleaning their hands*, *we eliminate it (the data)*. *We can use that sample once they eliminate the information*, *to avoid a problem there*, *right*? *As such*, *I don’t feel bad*, *and I’m not going to find out and it’s normal*, *right*? *The only thing I’m going to feel is like*, *ok*, *right; it’s a study*, *it was what I authorised*. *And that’s what I’m going to take with me*, *and after that*, *I don’t know what will happen*.*"*

(C22-DAR, Colombia, less than 25 years old, male, participant)

#### Information about future studies

While participants understood the phrase "can be used in this study or in other studies," they felt that the broad consent language in the IC did not provide sufficient information about reusing their data and samples. Participants expected to receive information about future studies and disagreed with the idea that they would not learn about future uses or users.

*"I am concerned that they will not give us information*, *they will not give us the results*, *we will not know anything*.*"*

(N16-MAE, Nicaragua, 25–34 years old, male, respondent’s guardian)

*"Imagine*, *something that my son’s blood has or his DNA begins to fly around the world and without knowing to whom and for whom*, *well*, *when one gives them [the samples]*, *let’s say to this group*, *well*, *one is confident that it is for something good*, *right*? *For an investigation about this little animal [the mosquito]*, *the consequences that it causes*. *But*…*we are going to take [the samples] to another country*, *to another city or other researchers*, *people that we don’t know*, *that are not working for research or other things*. *And that they do it without our permission*, *I don’t agree*.*"*

(P13- LDB, Colombia, 25–34 years old, female, respondent’s parent)

#### Fear of misuse

The lack of information about future use was related to some participants’ concern that samples could be misused to cause harm, including the development of new viruses or for cloning.

*"I would be a little hesitant because*…*look at what happened with COVID*, *which is said to have been something bad in laboratories*, *so it creates uncertainty and nerves*…*I would be worried that my son’s DNA would be used to create new viruses because*, *although it can be beneficial*, *it can also be bad because suddenly something can go wrong and it is possible that what we are currently experiencing could happen again*.*"*

(C16-YMA, Colombia, less than 25 years old, female, respondent’s parent)

*"*…*not [your research team] but rather other laboratories take [the sample] for other research that is not for medical purposes*.*"*

(C14-DMM, Colombia, 35–45 years old, female, respondent’s parent)

*"My concern is nothing more than that the samples are not occupied in what they really request*.*"*

(N2-MPL, Nicaragua, 35–45 years old, female, respondent’s parent)

#### Future use as a global public good

Among their reasons for sharing data and samples for future studies, interviewees mentioned the chance to improve scientific knowledge, vaccine development, and public health more generally.

*"I decided to share data and samples*. *As I said before*, *if it were not for the fact that there were people who allowed those samples to be preserved*, *we would not have vaccines or medicines that are doing us good right now*, *so I want to be a part of that good that can be done for humanity*.*"*

(C17-MSF, Colombia, over 45 years old, female, respondent’s parent)

*"What I am doing and what I am authorising is because I want a conclusion to be reached as such and to help so that it really becomes an investigation that can help society*. *Then*, *yes*, *I would give my authorisation that the [samples] can be used again for other future investigations*.*"*

(C22-DAR, Colombia, less than 25 years old, male, participant)

*"I agree with sharing data and samples because it would do others good*, *it would grow*, *it would really serve the research*, *the effort that was made*, *the purpose for which we can say my son donated blood*, *for studies to develop vaccines*. *So*, *I would like them to do many more things*, *many more*, *for the benefit of all*.*"*

(P8-IJG, Colombia, 35–45 years old, female, respondent’s parent)

*"I would feel lucky if a sample*, *say either my daughter’s or mine*, *was used to combat other diseases*.*"*

(N6-MPHM, Nicaragua-35-45 years old, female, respondent’s parent)

#### Sharing samples and data outside of the country

Most people supported sharing data and samples with research groups outside the country to expedite public health advances. Participants cited the COVID-19 pandemic to indicate how cross-national cooperation benefited public health through fast-tracking vaccine development.

*"I understand that the samples are not only going to be here in the country with the research teams*, *but they are also going to reach other countries*, *where I think that different points of view and different knowledge*, *well*, *it would be good because it brings together all that knowledge of each scientist*, *each researcher*, *then it is more feasible that together they can make a vaccine*, *like that of COVID-19*, *all the countries met and then together they looked to see how to do it and managed to get a vaccine*, *to prevent that disease*.*"*

(C16-YMA, Colombia, less than 25 years old, female, respondent’s parent)

*"I feel that we are giving a grain of sand to find a solution*, *a vaccine*…*We in this country do not have as much capacity to generate a vaccine*. *We are not like Cuba*…*not like other countries directly dedicated to finding a vaccine*, *like right now that they are finding a vaccine for COVID*.*"*

(N6-MPHM, Nicaragua, 35–45 years old, female, mother of a participant)

#### Moore Clause

The Moore Clause relates to a court case where a study participant who learned that tissues derived from his cells had been used in for-profit research sued the State of California to be compensated for that use [14]. The term "biological samples," used in the Moore Clause and throughout the template IC, was clear to most participants and was generally understood as blood samples. Participants did not say they wanted to receive a financial benefit from future research but expressed concern that a small group could monopolize the benefits from research.

*"Let’s say I contributed my samples to obtain [the Zika vaccine]*…*it [the vaccine] is going to be worth a lot*, *or well*, *it is going to be difficult to obtain it*. *It is not easy to reach for some people*, *and all this because this discovery*…*belongs to a group*, *so they benefit economically*, *and well*… *they are not interested in benefiting society*, *which is how I think it should be*. *And that was the reason as I told you at the beginning*, *that is why I gave the sample*.*"*

(C22-DAR, Colombia, less than 25 years old, male, participant)

### Benefit sharing

#### Expectation of direct benefit

Participants generally disagreed with the statement, "neither you nor the investigators will benefit directly or commercially from the research," because they associated study participation with their child’s receiving improved medical care, which may be related to their experience of receiving improved care through their participation in the cohort study.

*"Well*, *that the doctors come to the house and check the child as they have done so far*. *Because with dengue they do it*, *then with Zika they should do it*, *right*?*"*

(C19-MD, Colombia, 35–45 years old, female, respondent’s parent)

*"The visits they do*, *like the ones they do now for dengue*, *because those are good*. *When the child gets sick from anything*, *I call the lady*, *and the doctor comes to see the child*, *and they give him the medicine*, *which is very good*.*"*

(C21-NRS, Colombia, 35–45 years old, female, respondent’s parent)

*"My son is going to be more cared for by the doctors*, *just as they do now with dengue*, *the doctor comes when something happens to him*. *That’s good for my son too*.*"*

(P7-AJS, Colombia, over 45 years old, female, respondent’s parent)

*"So*, *they call him [the doctor] every time he [my son] is sick*. *[The doctors] are always looking out for my son*. *The doctors come when something happens to him*.*"*

(P9-SV Colombia, over 45 years old, female, respondent’s parent)

When people did understand that their child would not benefit directly from the reuse of their data or samples by future studies, they disagreed with this concept and felt that the future research should be used to improve their child’s care.

*"I disagree with [not receiving direct benefits]*. *I would feel good as long as they take the child into account to do studies*, *to continue looking at the diseases he has or his blood*, *viruses that he might have so that he will be well*.*"*

(P7-AJS, Colombia, over 45 years old, female, respondent’s parent)

#### Expectation of public health benefits

Several participants mentioned the importance of data and sample sharing to contribute to scientific or practice-related advances that would improve the lives of future generations.

*"Because*, *well*, *they could suddenly bring about something good*. *The cure for other things that arrive in the future*, *another epidemic or something*.*"*

(C19-MD, Colombia, 35–45 years old, female, respondent’s parent)

Participants understood the reuse of data and samples as a way to develop drugs and vaccines that could benefit humanity.

*"New generations are coming at the global level*, *and they will be the ones who are going to benefit from these new studies*. *This will be a bit of a relief because there are many diseases*, *not just Zika*, *not just dengue*, *there are many diseases that we do not have a cure for and it would be very good if this new generation is the beneficiary so that so many people don’t continue to die*.*"*

(N4-FVMD, Nicaragua, 25–34 years old, female, respondent’s parent)

#### COVID-19-related benefit sharing

Participants viewed participation in research and reusing data and samples from that research as an important contribution to epidemic response. Several participants highlighted the public health benefits of data and sample reuse in COVID-19 response.

*"I mean*, *the blood is going to be conserved*, *but to benefit the community*, *that is*, *to make the vaccine*, *for example*, *as we are right now with the COVID situation*, *we were waiting for the vaccine to free ourselves a little from that situation*, *so the same is with Zika*.*"*

(C21-NRS, Colombia, 35–45 years old, female, respondent’s parent)

*"*…*science*, *research should be*, *as such*, *within the public realm*, *right*? *For everyone*, *in other words*…*for the benefit of society*. *As is the case in what we are seeing right now [during the COVID-19 pandemic]*, *that it is also very necessary*.*"*

(C22-DAR, Colombia, less than 25 years old, male, participant)

Participants stressed the importance of ensuring access to vaccines or other preventative or treatment measures whose production was facilitated through the reuse of data or samples generated through their participation in research.

*"How about me giving blood for the COVID vaccine and not being able to get vaccinated?"*

(C18-NAP, Colombia, 25–34 years old, female, participant)

#### Data and sample sharing with the pharmaceutical industry

Participants expressed mixed views on sharing data and samples with industry. Some participants cited sharing data and samples with the pharmaceutical industry as a means to help develop medicines and vaccines.

*"I think it’s very good because that way more medicines suitable for the disease would be developed*.*"*

(N14-JNP, Nicaragua, 35–45 years old, female, respondent’s mother)

Other participants saw the use of data and samples by for-profit groups as a cause for concern. They associated sample reuse by industry with a lack of benefit sharing and the reuse of samples for nefarious purposes, including the development of novel viruses, which they saw as the origin of the COVID-19 pandemic and cloning.

*"[They are] advances for them*, *but that they are buying something that my daughter and I are contributing at no cost just to help the investigation and that they can come and buy without our knowledge and obtain an economic benefit*, *with something that we are donating without getting anything in return just to help*.*"*

(N13-MNRR, Nicaragua, 35–45 years old, female, respondent’s parent)

*"There*, *I would not be so happy because when they are shared with laboratories and they bring [outside of the country] medicine*, *injections*, *pills*, *etc*. *And they are commercial*, *they sell them and sometimes they are very expensive so that regular people cannot pay*. *Those with limited resources cannot pay*, *only the great millionaires that have enough money*, *not us poor people*.*"*

(N14-JNP, Nicaragua, 35–45 years old, female, respondent’s parent)

When we explained to participants that samples could be used to produce new drugs or vaccines, they were more likely to support sample sharing with industry. Still, they highlighted the need for equitable access and specified that new drugs or vaccines developed with their samples should be made available to research participants and their communities.

*"Let’s say I’m going to need [the vaccine] in case I’m infected and then what am I going to do*? *Let’s say*, *I contributed my samples to obtain [the vaccine] so*, *what am I going to do*? *It’s going to cost a ton of money or*, *well*, *it’s going to be difficult to get*. *It won’t be easily accessible to some people*, *and all this because that discovery now belongs to a group [the sponsors]*, *so they benefit financially*, *and well then*, *they would not be interested in benefiting society*, *which is how I think it should be*. *And that was the reason*, *as I told you at the beginning*, *that’s why I gave the sample*. *I believe that science and research should be for the benefit of everyone and should not be seen as an economic benefit*. *As such*, *because we are talking about health*, *we are not here to commercialise*, *so that is what I would like*.*"*

(C22-DAR, Colombia, less than 25 years old, male, participant)

### Trust in the research team

Most participants referenced trust in the research team (UIS in Colombia, Instituto de Ciencias Sostenibles in Nicaragua) as a critical driver of their decision to agree to the future use of their data and samples. This trust was based on their positive experiences with previous research studies and their close relationships with the cohort study staff, which relates to the long-term nature of these cohorts and cohort staff’s sustained efforts to ensure the cohorts benefit the source community. The trust was also associated with the perceived benefit of increased medical attention through their child’s participation in the cohort, including improved access to medical appointments and closer follow-up by the medical team.

*"Well*, *I trust*, *first in the XX university*… *and I know that they are not going to deliver (samples*, *data) to whichever research group or any laboratory because*, *yes*, *they must have a project*, *a study*, *it is not all [something that happens] overnight*. *Rather everything is well structured*, *right*? *They are professional and very ethical*.*"*

(C14-DMM, Colombia, 35–45 years old, female, respondent’s parent).

*"I already participated in that dengue study*…*and it went well for me because [the doctors] stayed there off and on*. *They were calling me*. *They were calling people asking how they were doing and all that*…*So I imagine that this new stage with Zika will be the same*.*"*

(C18-NAP, Colombia, 25–34 years old, female, respondent’s parent)

*"Until now they have behaved very well*, *they come and visit me*, *they look out for the child*. *So*, *it must be that the people from XX university are doing something good*.*"*

(C20-EM, Colombia, over 45 years old, female, respondent’s parent)

While participants expressed concerns about the possibility of misuse by future groups, this fear was balanced with their belief that the research team would not allow misuse to happen.

*"That’s the problem*, *you don’t know if it is for [a good purpose]*…*but in any case*, *if it’s for the benefit of the people*…*if it is to get some drug*, *some vaccine*, *well*, *yes*, *I would like other people to benefit as well*. *Well*, *in truth*, *I have faith that you will do things well*.*"*

(P6-ASU, Colombia, over 45 years old, female, respondent’s parent)

### Perception of the cognitive interview study

Most interviewees said they had gained knowledge about the data, sample sharing, and consent process through the cognitive interview. Participants noted that clarifying the IC in the cognitive interview improved their confidence in the study.

*"I had no idea that to carry out a study*, *they asked for so many permissions*, *such as taking the samples*, *storing them*, *to use them in the future*. *I had no idea because I thought that they did it without getting your permission*. *The fact that you are telling me that [the investigator] is going to do it for this or that*, *to analyse the genome*, *to analyse the DNA*, *gives me confidence*.*"*

(C17-MSF, Colombia, over 45 years old, female, respondent’s parent)

*"I have learned a lot I had never heard before*. *And I keep thinking about all that information because they should tell you when they will take your blood for those studies*. *I had never heard all those things*.*"*

(C18-NAP, Colombia, 25–34 years old, female, participant’s mother)

*"But look*, *at the moment*, *sometimes*, *it happens that you sign and you don’t even realise it*, *they can even take you to prison*, *because you don’t know at the moment when they are so rushed*, *so this becomes an experience we can learn from so that we pay attention to what we are doing*.*"*

(N15- LMMU, Nicaragua, 35–45 years old, female, respondent’s parent)

### Cross-group differences in the understanding and acceptance of broad consent for future use of data and samples

We did not find meaningful differences in understanding or agreement with key concepts between Colombia and Nicaragua or across participant gender, with the caveat that we could only conduct 11 interviews with men. Participants who had completed high school were better able to understand and describe abstract concepts, like genetic studies, than those who had not completed high school. In Colombia, we interviewed five men and five women, aged 19–21, who were themselves research participants. These participants had all finished high school, and three had post-secondary education. Both parents or legal guardians who had consented to their children’s participation and cohort participants themselves supported research participation. Like their parents or legal guardians, cohort participants expressed concern about sharing data or samples with for-profit companies that they saw as potentially monopolising research data.

*"I really would not have to complain because the only thing I’m doing is giving a contribution with a sample*, *helping them (*…*) that’s my contribution*…*But the researchers wouldn’t be able to get to those studies either*, *to those answers they want to get to*, *without my help and that of many others*, *you see*?*"*

(C24-MSB, Colombia, less than 25 years old, male, participant)

*"As I told you*, *I am an administrator*, *and I am studying administration*…*many times companies or especially groups*, *large groups*, *and in research there also have to be those groups*, *what they want to do is how to take advantage or*, *in such a case*, *be the first to arrive at an answer that no one has yet*… *and at the moment of having that*, *if*, *let’s say*, *there are several groups that have the same answers*, *then why do they unite themselves*? *To make an economic profit*, *then they raise prices*.*"*

(C22-DAR, Colombia, less than 25 years old, male, participant)

Cohort participants also questioned whether their parents understood the information communicated in the informed consent.

*"*…*When I participated in the study*… *I did not receive all this information*. *So*, *you make me think that my mom*, *for example*, *has no idea about these things*. *So*, *she just said "okay*," *but because she doesn’t know anything*, *you understand*?*"*

(C15-CCT, Colombia, less than 25 years old, male, participant)

#### Parental consent for sharing data or samples from their children

A few parents mentioned discussing data and sample sharing with their children.

*"I feel like we help other people*, *because maybe there are people who do not allow them to take their blood sample for research*. *In this case*, *I always take the consent with her [my daughter] if she decides to give the blood because she is already a lady and she also has her voice and her vote in saying yes or no*. *So*, *I feel that we are giving a grain of sand*, *that support on her part so that the investigations can proceed*, *regardless of her data or my personal data*.*"*

(N6-MPHM, Nicaragua-35-45 years old, female, respondent’s parent)

*"From the time [they started] the dengue study*, *I consulted [my daughter]*. *We have always given my children a little bit [of responsibility]*. *We have said that they make decisions from an early age*. *So*, *I am doing this not because I decided*, *but because she also [decided]*.*"*

(N17-JGG, Nicaragua, over 45 years old, male, respondent’s parent)

### Participant recommendations

#### Advice to future research participants

Most interviewees spoke about the importance of participating in research studies so that future generations could benefit from the results of these studies. They encouraged future research participants to look towards future public health advances rather than individual health or economic benefits.

*"You shouldn’t think about the individual*, *but rather about the group*, *that we must help each other*, *support each other as brothers and give the opportunity to a future patient*, *so to speak*. *So that they have a medicine that can be safeguarded more quickly than in the past*.*"*

(P5, Colombia, 25–34 years old, male, participant)

Participants suggested that future research subjects carefully read project documents to understand the study objectives, data, and sample management.

*"That first [the participants] read [the IC] well and that they understand the information that the researchers are giving them so that everything is understood*, *and they respond in the best way*.*"*

(C16-YMA, Colombia, less than 25 years old, female, respondent’s parent)

#### How researchers can improve broad consent

Participants asked that researchers provide more straightforward explanations about the research process in the IC. They said the technical language in the IC was not clarified in the written form or in the verbal explanation of the form.

*"I would tell them that everything they do seems very good to me and that*, *just like you researchers*, *we are people willing to collaborate so that we work together*. *But that things should be explained well*…*so that you can always believe them*.*"*

(P3-JS, Colombia, 35–45 years old, female, respondent’s parent)

Participants also suggested that the level of explanation and number of permissions in the IC could create concerns.

*"Well*, *I don’t explain myself*…*excuse me for the word as a Nicaraguan*, *that really makes me anxious*…*That you tell me that they are going to keep it a secret*, *like that my daughter after that study is going to take her out*, *they’re going to come and kidnap her*, *that’s what makes me understand*.*"*

(N17-JGG, Nicaragua, over 45 years old, male, respondent’s parent)

*"What I don’t understand is why they ask for so many authorisations*. *It seems that blood samples are delicate*… *but because they ask for so much authorisation and why they say so many things*.*"*

(C18-NAP, Colombia, 25–34 years old, female, participant)

#### Results of pilot of community-informed IC

We rewrote broad consent language in the IC template using the explanations of key phrases or concepts from research participants. Confusing phrases from the template IC and suggested language to improve understanding from community member interviews are included in S4 Table. Key differences between the community’s language and that of the UC Berkeley template included:

1. Separate sections for consent for 1) data and sample collection and preservation for the current study; 2) data and sample preservation and sharing for future studies, including the Moore Clause; 3) use of samples to derive genetic data for use in the current and future studies;
2. The clarification of the difference between participation in research and routine clinical care;
3. Specifying the type of sample (blood sample);
4. Clarifying the meaning of whole genome DNA or RNA sequencing using a "puzzle" as a visual analogy.

The substitution of the community’s language in the template IC and the additional clarifications resulted in a longer IC (one versus two pages in the template IC). Despite these additional clarifications, half of the eight participants in cognitive interviews with the community-informed IC still had trouble explaining genetic data in their own words. Participants in the community-informed IC cognitive interviews still associated study participation with their child’s receiving improved medical care and expected that incidental findings from future research would be shared.

## Discussion

We conducted cognitive interviews with research participants who had consented to their or their children’s participation in long-standing dengue-focused cohorts in Nicaragua and Colombia to explore their understanding of and agreement with the broad consent-related language in the UCB IRB template IC, which was required for use with human subjects research that involves genetic data and biospecimens at the time of the study [27]. The UCB template IC that we used stated that incidental findings would not be communicated and participants almost universally disagreed with this statement and expressed the desire to be informed about incidental findings. How to inform participants about incidental findings is an emerging research area that requires further research [28, 29].

Participants did not understand that they would not be informed about the future uses or users and disagreed with the idea that they would not be informed about incidental findings. They wanted to know more about the types of future users and to learn about future studies’ results. While participants felt that the UCB template did not provide enough information, participants that received the template with the community-informed language felt that there was too much information. In a few cases, the increased number of explanations in the revised template had the unintended consequence of making interview participants worry about why so many explanations were given.

Participants saw data and sample reuse as an important contribution to open science but felt that they did not have enough clarity on future uses or users. As with the UCB template IC, many ICs do not specify which groups will be responsible for making decisions about the future use of data or samples [30]. Investigators often straddle the line between broad and blanket consent because they would like to maximize the utility of the data and samples and may be unable to envision the possible uses of the data or samples. In the push for open data, many prominent global health journals and funders now require the publication of study data in a repository [31, 32], resulting in a range of uses more closely aligned with blanket than broad consent. Some participants expressed concerns about using data or samples for commercial purposes or by bad actors. Participants cited sample reuse for illicit purposes as a potential cause of the COVID-19 pandemic, which was ongoing during this study.

While regulatory concerns around data and sample sharing focus on the threat of reidentification [33], participants generally did not cite reidentification of themselves or their children as a concern when discussing data and sample reuse. Participants’ focus on securing benefits for the community and for science rather than the possibility of reidentification suggests that benefit sharing is more important to research participants than the threat of reidentification and potential consequences thereof.

Study participants’ primary motivations for consenting to data and sample sharing as part of their child’s participation in the original cohort study were trust in the study team related to the long-standing presence of the study staff and cohort in their community and effective benefit sharing at the individual, community, or global levels. Parents felt they had better access to medical staff through participation in the study, the study communicated results to individuals and the local or national health authority, and saw the cohort as otherwise benefiting their community. They felt that sharing their data and samples could enable faster development of vaccines, citing the research response to COVID as an example. These findings are supported by prior work that found health research participants see data and sample sharing as a way to improve their care, improve care for people who share their condition, and contribute to Open Science [34, 35].

IC is critical to the ethical conduct of research (principle of respect for persons) and is vital for enrolment and continuity of participation in cohort studies [3, 7]. A 2015 systematic review of qualitative and quantitative studies published prior to 2013 found a number of issues related to understanding of IC for participation in clinical trials [36]. More recent studies that evaluated understanding of IC for clinical trials applied a quantitative score to assess IC readability [37, 38], post-IC quizzes with research participants [39], surveys [40], or semi-structured interviews [41] and similarly found that IC language was not well understood. Only one study we identified evaluated potential understanding of the language used to convey broad consent. That study, a textual analysis of IC forms rather than human subjects research, found that most of the 29 ICs reviewed had a high Flesch score, suggesting research participants would have had difficulty understanding the broad consent text [37]. Most of these studies were conducted in high-income settings and could not identify studies that evaluated understanding of IC for observational research or understanding of broad consent for future use specifically, suggesting in the need for more research in this area.

Investigators and ERCs are in a difficult place where the need to explain complex concepts in accessible language competes with making the consent form as concise as possible. One way forward could be to reposition the consent process as an open conversation focused on ensuring participant understanding rather than a list of complicated, legalistic points focused on preventing lawsuits or demonstrating institutional adherence to complex legal structures (e.g., the European General Data Protection Regulation; GDPR).

While the concept of future use was well understood, most participants had difficulty understanding scientific or medical language in the broad consent section of the template IC, including genetic studies, biological samples, de-identified data, and incidental findings. Participants reported that the UCB IC was similar to ICs they received for other studies, which also included difficult-to-understand technical and medical language that they said was generally not clarified in either the written IC or the verbal explanation.

Despite the additional explanations, the use of samples to conduct genetic studies and how genetic data are then shared was not well understood, suggesting the need for building research participants’ understanding of this concept through alternative strategies that have been successful in clarifying genetic studies and sharing genetic data, like video clips [42].

The collection and reuse of genetic data are sensitive and challenging to explain. Previous studies indicate that ICs for genetic research do not adequately explain what will happen with incidental findings, sample management, and sample and data governance [43]. IC provides potential participants with information related to the ethical principles of beneficence, minimizing the harms and maximizing the benefits of participating in research, justice, the alignment of the research with the values of the community, and engaging the community in learning about the results of the study and benefiting from the research. These studies remind us that, besides ensuring that participants understand broad consent for future use, the IC must appropriately address these ethical concerns.

While participants better understood the community-informed IC, the perception that study participation results in improved care at the individual level highlights the challenge of navigating the threat of coercion and the need to engage in appropriate benefit sharing when conducting research in low-resource settings.

The clear preference for learning more about future users and uses, being told about incidental findings, and being able to access the results of future studies, means that cohort participants in Nicaragua and Colombia expected that their shared data and samples could be linked to them so that they could be re-contacted. This finding posits community preferences for learning about future studies against the pressure on research teams to anonymize (remove the possibility of reidentifying a participant) rather than pseudonymize (remove direct identifiers and assign a code to participant data to enable reidentification if needed) participant-level data to assuage institutional concerns around GDPR compliance.

### Strengths & limitations

This study has several strengths. This is the first study to use cognitive interviews to assess participants’ understanding of broad consent for future use-related language. We tested participants understanding of broad consent language from the IC template from a large public university that conducts a high volume of biomedical studies in high- and LMICs. While the interpretation of broad consent-related language likely varies across populations and contexts, the finding that participants did not sufficiently understand the language indicates that universities and research teams should consider using qualitative approaches to develop or evaluate the language in IC forms.

This study has some limitations. Ideally, the project interviewers would have been source community members to prevent hierarchical power dynamics during the interviews. Because the project interviewers had long-standing relationships with cohort members, they could develop a good rapport. Additionally, we could not include enough male participants to allow for cross-gender comparisons of responses. In both settings, women are generally responsible for taking care of educational and health-related issues for their children and were most often responsible for consenting to their children’s participation in the source cohort studies. Ideally, the cognitive interviews would have been conducted in person, but we conducted them by phone in Colombia, in keeping with local regulations during the COVID-19 pandemic. We compared field notes between Colombia and Nicaragua and did not find substantial differences in the flow of the cognitive interview session between in-person and phone-based interviews. This finding is in keeping with other work that found that the rapport needed for qualitative research could be built by phone [44].

### Recommendations for research and practice

The ethics community has moved away from the paradigm of ERCs in high-resourced settings evaluating the ethics of research conducted in low-resource settings to mandating in-country ethics review for multi-country studies. While this move can help ensure that local values that shape interpretations of shared ethical principles are included in the IC language and structure, local ERCs may be reluctant to change the language of template ICs from ERCs that review the protocols for multi-country studies, which are often managed by researchers based in high-income settings. A 2012 qualitative study with 46 representatives from 34 US IRBs found that IRBs members reported lack of knowledge of local context and how the ethical principles of autonomy, justice, and proportionality are considered in different contexts as major challenges [45]. The same study found that US-based ERCs’ fear of "worse contributions scenarios" related to research management sometimes led them to paternalistic interactions with ERCs in LMICs [45]. While ERCs need to ensure continuity in adherence to widely accepted ethical principles across studies and countries, context is important. ERCs should allow investigators, which need to include representatives from the community participating in the study, sufficient leeway to tailor the consent language and content to reflect community values and preferences.

Participants stressed benefit sharing, providing information on future uses and users, and maximizing the utility of data and samples over privacy concerns. Some legal frameworks related to data and sample reuse, like GDPR, complicate efforts to share data and samples directly or through their interpretation. Researchers or institutions concerned about GDPR compliance may decide to anonymize rather than pseudonymize their data given that sharing anonymous data falls outside of GDPR. When investigators share anonymous data, data where the probability of the reidentification of individual participants approaches zero, researchers cannot inform participants about future uses and users of their data or samples or the incidental findings that arise from those studies. Policy makers should consider whether the current legal framework for data and sample sharing, which is rooted in concerns about the privacy-related harms of data and sample sharing rather than the equitable distribution of benefits from research, aligns with research participant values.

## Conclusion

This study adds to the growing body of evidence suggesting that IC is not well understood and is the first human subjects research on how broad consent is understood. We found that participants’ trust in the research team, rather than the explanation provided in the IC, was the most important foundation for their support for data and sample sharing. Trust in teams and systems, rooted in effective benefit sharing and close relationships between researchers and research participant communities, may be more closely related to data and sample sharing-related decision making than IC, which participants may not well understand. While participants were concerned about sharing data and samples with for-profit groups, they generally expected that their data and samples would be reused. They wanted to learn about how data and samples were reused and any incidental findings. Researchers should consider sharing pseudonymized rather than anonymized data to allow for the communication of incidental findings and ensure participants can learn about the results of future studies that use their data or samples. Cognitive interviews can help ensure that language around broad consent for future use of data and samples is well understood. In addition to meeting the ethical principle of respect for persons, ensuring understanding of broad consent for future use fosters trust in the research process and data or sample sharing more broadly.

## Supporting information

S1 Table Relevant sections of the University of California Berkeley informed consent. (DOCX)

S2 Table Codebook. (DOCX)

S3 Table Supporting quotes in original Spanish and English translation. (DOCX)

S4 Table Confusing phrases in template informed consent and suggested language. (DOCX)

S1 Text Interview guide that evaluates understanding of language from the Spanish translation of the University of California at Berkeley Spanish-language template informed consent for biomedical studies. (DOCX)

S2 Text Interview guide that evaluates understanding of language from the Spanish translation of the University of California at Berkeley Spanish-language template informed consent for biomedical studies updated with language from explanations from research participants. (DOCX)

S3 Text PLOS Inclusivity in global research questionnaire. (DOCX)
