# Supplementary material for: "How about me giving blood for the COVID vaccine and not being able to get vaccinated?" A cognitive interview study on understanding of and agreement with broad consent for future use of data and samples in Colombia and Nicaragua
Source: PLOS Glob Public Health. 2023 May 17;3(5):e0001253. doi: 10.1371/journal.pgph.0001253 (PMC10191364; doi:10.1371/journal.pgph.0001253)
Supplement: S1 Table — (DOCX) [file pgph.0001253.s001.docx]

**S1 Table. Relevant sections of the University of California Berkeley informed consent [1]**

| Genetic Data | We would like to have additional permission to store your child's clinical information, blood samples and DNA for future genetic studies. Genetic studies examine the genetic information (represented by DNA) passed on from parents to children. We will specifically be looking for genes that might make people more or less likely to develop severe diseases caused by arboviral infections and other genes related to health status…The DNA samples and clinical information will be sent, without your child's name, to one of our research partners and may be sent to other research partners collaborating with our laboratory in the future. Any extra samples will be stored in the CNDR in Nicaragua or in our laboratory at the University of California, Berkeley. The DNA samples and information will be used for research, and such use could possibly result in inventions and discoveries that could become the basis for new commercial products, diagnostic tests, or therapeutic agents. However, while neither you and your child nor the investigators will benefit directly or commercially from this part of the research, future generations may benefit from the knowledge that is gained. Your child's DNA samples and clinical information will be made available to the Broad Institute and other researchers only if you agree to this procedure. Your child's samples and information will be assigned a unique identification number. They will not contain any information (such as your child's name or address) that could identify you or your child. |
| --- | --- |
| Incidental findings | Clinically relevant research results, including individual research results, will not be disclosed to subjects. |
| Broad consent for future use | Identifiers might be removed from the identifiable private information or identifiable biospecimens. After such removal, the information or biospecimens could be used for future research studies or distributed to other investigators for future research studies without additional informed consent from the subject or the legally authorised representative. |
| Moore Clause | Biospecimens (such as blood, tissue, or saliva) collected from your child for this study and/or information obtained from your child's biospecimens may be used in this research or other research and shared with other organisations. You will not share in any commercial value or profit derived from the use of your child's biospecimens and/or information obtained from them. |
| Benefit sharing | There will be no direct benefit to you from participating in the study.  However, this study will allow us to learn more about [procedures/ drugs/ interventions/ devices], and we hope that this information will help in the future treatment of individuals with [. . . /conditions like yours]. |

1. Template Consent Form - Biomedical Study Berkeley, California: University of California at Berkeley; [cited 2022 March 31]. Available from: <https://cphs.berkeley.edu/CF-Template_Biomed.docx>.
