# Supplementary material for: "How about me giving blood for the COVID vaccine and not being able to get vaccinated?" A cognitive interview study on understanding of and agreement with broad consent for future use of data and samples in Colombia and Nicaragua
Source: PLOS Glob Public Health. 2023 May 17;3(5):e0001253. doi: 10.1371/journal.pgph.0001253 (PMC10191364; doi:10.1371/journal.pgph.0001253)
Supplement: S2 Table — (DOCX) [file pgph.0001253.s002.docx]

**S2 Table. Codebook**

| **Themes** | **Codes** |
| --- | --- |
| Trust in the research team | Trust in the research team |
| Recommendations for broad consent language and process | Recommendations for future research participants |
|  | Recommendations for researchers |
| Perceptions of governance for data and sample reuse and future users | In favor or against sharing data or samples with non-profit groups |
|  | In favor or against sharing data or samples with industry/for profit groups |
|  | Types of worries related to sample sharing |
|  | Motivations for support of data sharing |
|  | Motivations against data sharing |
| Understanding of key concepts in broad consent section | Direct identifiers |
|  | Confidentiality |
|  | Confusion between data and samples |
|  | Uses of information |
|  | Sample storage, biobanking |
|  | Reuse of samples |
|  | Confusion related to future use of samples |
|  | Sharing samples |
|  | Concept of a sample |
|  | Concept of research studies |
|  | National research institutions |
|  | International research institution |
|  | Commercial products, diagnostics, therapeutic agents |
|  | Resultados de la investigación e individuales (clinicos) |
|  | Genome, genetic data, genetic studies |
| Benefit sharing | Direct benefits |
|  | Public health benefits |
