# Supplementary material for: "How about me giving blood for the COVID vaccine and not being able to get vaccinated?" A cognitive interview study on understanding of and agreement with broad consent for future use of data and samples in Colombia and Nicaragua
Source: PLOS Glob Public Health. 2023 May 17;3(5):e0001253. doi: 10.1371/journal.pgph.0001253 (PMC10191364; doi:10.1371/journal.pgph.0001253)
Supplement: S3 Table — (DOCX) [file pgph.0001253.s003.docx]

**S3 Table. Supporting quotes in Spanish and English, organized by theme**

| **Theme (Participant information)** | **Representative quote - Spanish** | **Representative quote - English** |
| --- | --- | --- |
| **Genetic studies** |  |  |
| (P6-ASU, Colombia, more than 45 years old, female, participant's mother) | “Estudios genéticos? ¿Que van a buscar genes de otras personas en la sangre de mi hijo? Entiendo más o menos eso” | “Genetic studies? Are they going to look for genes from other people in my son's blood? That's more or less what I understand." |
| (N20-MSMR, Nicaragua, more than 45 years old, female, respondent's parent) | “estudios genéticos son las muestras que piden para los padres para saber si el hijo es suyo o no.” | "Genetic studies are the samples they ask for the parents to know if the child is theirs or not" |
| **Commercial products** |  |  |
| (P6-ASU, Colombia, 35-45 years old, female, respondent's parent) | ”Pues las pruebas de diagnóstico son las del laboratorio, no?  Lo otro no lo entiendo.” | “Well, the diagnostic tests are those of the laboratory, right? I don't understand the other.” |
| **Biological samples** |  |  |
| (C22-DAR, Colombia, less than 25 years old,  male, participant) | “Muestras biológicas, apenas me dijo biológicas yo entendí algo familiar, no tengo ni la menor idea que responder.” | "Biological samples, as soon as you told me biological I understood something familiar, I don't have the slightest idea what to answer." |
| **Diagnostic tests** |  |  |
| (P6-ASU, Colombia, 35-45 years old, female, respondent's parent) | “Pues las pruebas de diagnóstico son las del laboratorio, no?  Lo otro no lo entiendo, lo de… eso… no, no sé qué sea eso” | “Well, the diagnostic tests are those of the laboratory, right? I don't understand the other.” |
| **Sample storage** |  |  |
| (C22-DAR-Colombia-  less than 25 years old,  male, participant) | “...así como los bancos de sangre deberían tenerlas en una bodega, en un cuarto totalmente frío… no se vaya a dañar.” | "...just like a blood bank, they should keep them in a warehouse, in a totally cold room…so that the samples are not damaged." |
| (N6-MPHM, Nicaragua, 35-45 years old, female, respondent's parent) | “....es donde almacenan muestras de sangre o los tipos de sangre, cuando las personas necesitan y creo que para eso son las donaciones y las reciben por cualquier emergencia.” | “....it is where they store blood samples or blood types, when people need them and I think that is what donations are for and they receive them in case of an emergency.” |
| (N19 JALC, Nicaragua, 35-45 years old, male, father of a participant) | "Digamos un mal es en el caso que digamos que ella padezca de una enfermedad crónica y venga usted y digamos la institución vino y ocuparon la muestra de mi hija y la persona que recibió esa sangre, se enfermó." | “Let's say one bad thing is in the case that, let's say she suffers from a chronic disease and you come and, let's say, the institution came and they took the sample from my daughter and the person who received that blood, got sick.” |
| **Confidentiality** |  |  |
| (P11-EYP, Colombia, 35-45 years old, female,respondent's parent) | “Que van a compartir mi sangre, y pues, en realidad, yo pensaría que eso estaría mal porque uno tiene que saber…Puede ser que hagan algo malo o que haya alguna enfermedad y uno no sepa, y después uno está enfermo y, y ni se entere. Por eso estaría mal” | “That they are going to share my blood, and well, in actuality, I would think that would be wrong because you have to know….It could be that they do something wrong or that there is some illness and you dont know about it, and afterwards you are sick and, and you don’t even find out. That’s why it would be bad." |
| (N17-JGG, Nicaragua, more than 45 years old, male, respondent's parent) | “Estoy preocupado porque me dice que todo dato se va a borrar. Hasta cierto punto como que esa idea no estoy tan de acuerdo, porque si es muy lógico que no hay ninguna retribución. Pero por lo menos digo yo que si salís pegado en algo por lo menos se te avise.” | “I am worried because this tells me that all data will be erased. To a certain extent I do not agree with that idea, because it makes sense that there won't be any negative consequences. But at least I say that if you get hit [you were infected] you should be informed.” |
| (N5-CMAZ, Nicaragua, 35-45 years old, female, respondent's parent) | “En algunos casos me siento bien y en otros casos no porque siempre está la idea que ¿Qué es lo que va  a pasar con la muestra? Y si realmente la información será confidencial,  lo que más temor le da a uno como padre es que le vaya a suceder algo al niño, por nosotros estar dando tanta información de él.” | “In some cases I feel good and in other cases not because there is always the idea: what is going to happen to the sample? And if the information is really going to be confidential, what one fears the most as a parent is that something is going to happen to the child, because we are giving so much information about him.” |
| **Clinically relevant results** |  |  |
| (P13 LDB, Colombia, 25-34 years old, female,respondent's parent) | “Clínicamente relevantes es cuando los resultados son malos, cuando sale algo inesperado, algo que sea relevante del niño pues.” | "Clinically relevant is when the results are bad, when something unexpected comes out, that would be relevant to the child." |
| **Non-communication of incidental findings** |  |  |
| (N17 JGG, Nicaragua, more than 45 years old,male, respondent's parent) | “Yo lo miro malo, que se queden con ese resultado, si en algún caso hay algún tipo de enfermedad que ellos descubran, aparte del que estamos haciendo, porque el estudio de muestra de la sangre no es solo para Zika, me estás diciendo que va haber otro tipo de exámenes o estudios que se le van hacer a las muestras, entonces si usted me dice que no va haber ningún resultado individual o que le vayan a decir a la persona no me parece bien, en ese sentido le pongo el caso cuando uno es donante de sangre.” | “I see it as bad that they keep that result, if in any case there is some type of disease that they discover, apart from what we are doing, because the blood sample study is not only for Zika, you are telling me that there will be other types of exams or studies that are going to be done on the samples, then if you tell me that there is not going to be any individual result or that they are going to tell the person I don't think it's right, in that sense I put the case when a person is a blood donor.” |
|  |  |  |
| (N12- MMAG, Nicaragua, 35-45 years old, female, respondent's parent) | “Como es para investigación del Zika a futuro, la preocupación era que encontraran algo y dijeran: ¡En la persona tal encontramos en sus genes  algo! o que se yo, algún tipo de enfermedad y lo publiquen y que se revele el nombre de la persona.” | "Since it's for future Zika research, the concern was that they would find something and say, 'In such-and-such a person we found something in their genes!' or what do I know, some kind of disease and publish it and the person's name would be revealed." |
| (P13-LDB, Colombia, 25-34 years old, female, respondent's parent) | “Yo creo que tenemos derecho a saber si de pronto en ese estudio salió algo que pueda afectar a mi hijo en su salud.” | "I think we have the right to know if something suddenly came out of that study that could affect my son's health.” |
| (P4-JS, Colombia, more than 45 years old, female, respondent's parent) | “…si sale algo relevante pues sí me gustaría que me lo dijeran.” | “...if something relevant comes out, then yes, I would like them to tell me.” |
| (NPILOTO2-ESLO, Nicaragua, more than 45 years old,  female, respondent's parent) | “yo estoy consciente que usted va hacer el estudio, tal vez así sacan una vacuna para que se cure ese Zika. Si el niño tiene algo relevante no me vas a decir y eso me preocupa.” | “I am aware that you are going to carry out the study, perhaps that way they will produce a vaccine to cure Zika. But if the child has something relevant, you are not going to tell me and that worries me.” |
| (N3-CCBB, Nicaragua, 35-45 years old, female,respondent's parent) | “Mi manera de pensar era de que si pasaría algo anormal en el tipo de sangre de ella se iba a comunicar, pero me estás diciendo que no serán revelados” | "My way of thinking was that if something abnormal happened in her blood type, it would be communicated, but you are telling me that it would not be revealed. Practically, [the information] is for you and that's it. Is that right?" |
| **Broad consent for future use** |  |  |
| (C22-DAR, Colombia, less than 25 years old,  male, participant) | “Pues ya autoricé para que la usen para lo del Zika y también autorice que se eliminen los datos una vez, entonces, pues bueno autorizo ¿no? porque es como un formato que se está llenando ¿no? Para poderlo rotar, para podérselo dar a otros investigadores, entonces es como que eso ya ahí, como que se están limpiando las manos, no se como decirlo con otra frase…mmm como se limpian las manos…es como que… lo eliminamos y podemos usar esa muestra una vez eliminemos la información, una vez se haya sacado todo, entonces para evitar ahí un problema ¿no? Como tal no me siento tampoco mal y no me voy a enterar y pues normal ¿no? Yo lo único que voy a sentir es como bueno listo, es un estudio, fue lo que autorice y eso es lo que me voy a llevar yo, y ya después de eso no sé qué va a suceder.” | “Well, I already authorized them to use it for the Zika study and I also authorized the deletion of the data once, so, well, I authorize it, right? because it is like a format that is being filled, right? In order to be able to rotate it, to be able to give it to other researchers, then it is as if it is already there, as if they are cleaning their hands, we eliminate it (the data) and we can use that sample once they eliminate the information, to avoid a problem there, right? As such I don't feel bad and I'm not going to find out and it's normal, right? The only thing I'm going to feel is like, ok, right, it's a study, it was what I authorized. And that's what I'm going to take with me, and after that I don't know what's going to happen.” |
| **Receiving information about future studies** |  |  |
| (N16-MAE, Nicaragua, 25-34 years old, male, respondants´tutor/guardián) | “Me preocupa que no nos van a dar información, no nos van a dar los resultados a nosotros, no vamos a saber nada.” | “I am concerned that they will not give us information, they will not give us the results, we will not know anything.” |
| (P13- LDB, Colombia, 25-34 years old, female, respondent's parent) | “Imagínate, algo que tenga mi hijo su sangre o su ADN empiece por ahí a volar por todo el mundo y sin saber a quien y para quien, pues, cuando uno las entrego [las muestras), digamos a este grupo, pues, uno va confiado en que es para algo bueno, ¿no? Para una investigación sobre este animalito [el mosquito], las consecuencias que el hace. Pero ya más allá de que vamos a ir a llevárselas a otro país, a otra ciudad o a otros investigadores, gente que uno no conoce, que no están trabajando para investigación o para otras cosas, y que lo hagan sin el permiso de uno, no me parece.” | "Imagine, something that my son's blood has or his DNA begins to fly around the world and without knowing to whom and for whom, well, when one gives them [the samples], let's say to this group, well, one is confident that it is for something good, right? For an investigation about this little animal [the mosquito], the consequences that it causes. But beyond the fact that we are going to take them to another country, to another city or to other researchers, people that we don't know, that are not working for research or for other things, and that they do it without our permission, I don't agree." |
| **Fear of misuse of data or samples by future users** |  |  |
| (C16-YMA, Colombia, less than 25 years old, female, respondent's parent) | “Estaría un poco indecisa porque…mire lo que paso con el COVID que se dice que fue algo mal implementado en los laboratorios, entonces se crea incertidumbre y nervios… me preocuparía que se use el ADN de mi hijo para crear nuevos virus, porque aunque puede ser de beneficio también puede ser malo porque de pronto algo pueda salir mal y es posible que se vuelva a vivir lo que estamos viviendo en la actualidad.” | "I would be a little hesitant because…look at what happened with COVID which is said to have been something bad in laboratories, so it creates uncertainty and nerves...I would be worried that my son's DNA would be used to create new viruses, because although it can be beneficial it can also be bad because suddenly something can go wrong and it is possible that what we are currently experiencing could happen again." |
| (C14-DMM, Colombia, 35-45 years old, female,respondent's parent) | “...no son cosas de ustedes si no de pronto los otros laboratorios, que la lleven que sea como para otras investigaciones si, que no sea con fines médicos” | “...not [your research team] but rather other laboratories take [the sample] for other research that is not for medical purposes.” |
| (N2-MPL, Nicaragua, 35-45 years old, female, respondent's parent) | “Mi preocupación es nada más es que las muestras no sean ocupadas en lo que realmente solicitan.” | "My concern is nothing more than that the samples are not occupied in what they really request." |
| **Future use as a global public good** |  |  |
| (C17-MSF, Colombia, over 45 years old, female, participant's mother). | “Me hace tomar la decisión [de compartir datos y muestras] lo que le decía anteriormente, de no ser porque hubo personas que permitieron que se conservaran esas muestras, no tendríamos vacunas o medicamentos que en este momento nos están haciendo bien, entonces quiero ser partícipe de ese bien que se le puede hacer a la humanidad” | “I decided to share data and samples, as I said before, if it were not for the fact that there were people who allowed those samples to be preserved, we would not have vaccines or medicines that are doing us good right now, so I want to beColombia, more than 45 years old, female, respondent's parent) |
| (C22-DAR, Colombia, less than 25 years old,  male, participant) | “Yo lo que estoy haciendo y estoy autorizando es porque quiero que se llegue a una conclusión como tal y servir de ayuda para que realmente se llegue a una investigación que pueda ayudar a la sociedad. Entonces, sí daría mi autorización de que las puedan utilizar y puedan llevarlos a otras investigaciones futuras.” | “What I am doing and what I am authorizing is because I want a conclusion to be reached as such and to help so that it really becomes an investigation that can help society. Then, yes, I would give my authorization that the [samples] can be used again for other future investigations.” |
| (P8-IJG, Colombia, 35-45 years old, female, respondent's parent) | “Estoy a favor de compartir datos y muestras  porque haría bien a los demás, crecería, serviría realmente la investigación, el esfuerzo que se hizo, el propósito con la sangre que digamos dono mi hijo, para estudios, para realizar vacunas, entonces sí me gustaría que se hicieran muchas cosas más, mucho más, para el beneficio de todos.” | “I agree with sharing data and samples because it would do others good, it would grow, it would really serve the research, the effort that was made, the purpose for which we can say my son donated blood. For studies, to develop vaccines. So I would like them to do many more things, many more, for the benefit of all.” |
| (N19 JALC, Nicaragua, 35-45 years old, male, father of a participant)    (N6-MPHM, Nicaragua-35-45 years old, female, respondent's parent) | “me sentiría dichoso si una muestra digamos ya sea la de mi hija o sea mía se usara para bienes futuros, bienes futuros es digamos, para combatir otras enfermedades, me sentiría bien, si supieran que de esa misma muestra se pudiese usar para  combatir, ya sea cualquier enfermedad digamos como Dengue, Malaria, sería fenomenal.” | “I would feel lucky if a sample, say either my daughter's or mine, was used to combat other diseases, I would feel good if they knew that the same sample could be used to combat either any disease, say Dengue, Malaria, it would be great.” |
| **Sharing data and samples outside of the country** |  |  |
| (C16-YMA, Colombia, less than 25 years old, female, respondent's parent) | “Entiendo que las muestras no solamente van a estar aquí en el país con los equipos de investigación sino que también van a llegar a otros países, en donde pues pienso yo que diferentes puntos de vista y diferentes conocimientos, pues sería bueno porque reúne todo ese conocimiento de cada científico, cada investigador entonces es más factible que entre todos pongan un grano de arena y puedan hacer una vacuna, como por lo menos la del Covid-19 que fue otra cosa también igual ¿sí?, todos los países se reunieron y eso entonces entre todos miraron a ver cómo hacer y lograron sacar una vacuna, para prevenir esa enfermedad.” | “I understand that the samples are not only going to be here in the country with the research teams, but they are also going to reach other countries, where I think that different points of view and different knowledge, well, it would be good because it brings together all that knowledge of each scientist, each researcher, then it is more feasible that together they can make a vaccine, like that of Covid-19, all the countries met and then together they looked to see how to do it and managed to get a vaccine, to prevent that disease.” |
| (N6-MPHM, Nicaragua, 35-45 years old, female, mother of a participant) | “Siento que nosotros estamos dando un granito de arena para encontrar una solución, una vacuna, porque pienso que nosotros en el país no tenemos tanta capacidad para generar una vacuna, no somos como por ejemplo Cuba…no como otros países desarrollados que se dedican directamente para encontrar una vacuna, como ahorita que están encontrando vacuna para el Covid.” | “I feel that we are giving a grain of sand to find a solution, a vaccine, because I think that we in this country do not have as much capacity to generate a vaccine. We are not like Cuba…not like other countries that are directly dedicated to finding a vaccines,  like right now that they are finding a vaccine for Covid.” |
| **Expectation of direct benefit** |  |  |
| (C19-MD, Colombia, 35-45 years old, female, respondent's parent) | “Pues que vengan a la casa [los médicos]  y revisen al niño como lo han hecho hasta ahora. Porque con el dengue si lo hacen, entonces con el Zika lo deberían hacer no?” | “Well, that the doctors come to the house and check the child as they have done so far. Because with dengue they do it, then with Zika they should do it, right?” |
| (C21-NRS, Colombia, 35-45 years old, female, respondent's parent) | “Esas visitas que hacen, como las que ahora le hacen por el dengue, porque eso está bien. Cuando el niño se pone enfermo por cualquier cosa,  yo llamo a la señora y viene el médico a ver al niño y le dan el medicamento, eso es muy bueno.” | “The visits that they do, like the ones they do now for dengue, because those are good. When the child gets sick from anything, I call the lady and the doctor comes to see the child and they give him the medicine, that is very good.” |
| (P7-AJS, Colombia, more than 45 years old, female, respondent's parent) | “Él va a estar más cuidado así como hacen ahora con lo del dengue, que viene el medico cuando algo le pasa. Eso es bueno para mi hijo también” | “My son is going to be more cared for by the doctors, just as they do now with dengue, the doctor comes when something happens to him. That's good for my son too." |
| (P9-SV Colombia, more than 45 years old, female, respondent's parent) | “Pues es que a él me lo llaman cada vez que se enferma… siempre están pendientes… los doctores vienen cuando le pasa algo.” | “So, they call him everytime he is sick,  [the doctors] are always looking out for my son, the doctors come when something happens to him.” |
| (P7-AJS, Colombia, more than 45 years old, female, respondent's parent) | “Pues no, en eso es que no estoy de acuerdo. Yo me sentiría bien siempre y cuando lo tengan en cuenta para hacerle estudios, para seguirle mirando las enfermedades que tenga o la sangre, virus que vayan a tener, para que esté bien” | "Well, no, I don't agree with [not receiving direct benefits]. I would feel good as long as they take the child into account to do studies, to continue looking at the diseases he has or his  blood, viruses that he might have, so that he will be well." |
| **Expectation of benefit to public health** |  |  |
| (C19-MD, Colombia, 35-45 years old, female, respondent's parent) | “Porque pues podrían sacar de pronto algo bueno. Que llegue otras enfermedades y saquen un…de pronto la cura para a futuro otras cosas que lleguen, otra epidemia o algo.” | "Because, well, they could suddenly bring about something good. The cure for other things that arrive in the future, another epidemic or something." |
| (C21-NRS, Colombia, 35-45 years old, female, respondent's parent) | “o sea, la sangre va a ser conservada, pero para beneficiar la comunidad, o sea, para hacer la vacuna, por ejemplo, como estamos ahorita con la situación del COVID, estábamos esperando la vacuna para liberarnos un poco de esa situación, entonces lo mismo es con lo del zika”. | “I mean, the blood is going to be conserved, but to benefit the community, that is, to make the vaccine, for example, as we are right now with the COVID situation, we were waiting for the vaccine to free ourselves a little from that situation, so the same is with Zika.” |
| (C22-DAR, Colombia, less than 25 years old,  male, participant) | “...es que la ciencia, las investigaciones pues deben ser como tal al alcance público, ¿no? Para todos mejor dicho…para el beneficio sobre todo de la sociedad. Caso tal como lo estamos viendo ahorita que es muy necesario también” | “...science, research should be, as such, within the public realm, right? For everyone, in other words…for the benefit of society. As is the case in what we are seeing right now [during the COVID-19 pandemic], that it is also very necessary.” |
| (N4-FVMD, Nicaragua,25-34 years old, female, respondent's parent) | “Vienen nuevas generaciones a nivel mundial y serán los que se van a beneficiar de estos nuevos estudios. Lo cual será un poco de alivio porque hay muchas enfermedades, no solo el Zika, no sólo el Dengue, hay muchas enfermedades de las cuales no se ha encontrado cura y sería muy bueno que esta nueva generación sea la beneficiada para que ya no sigan muriendo tantas personas” | “New generations are coming at the global leevel and they will be the ones who are going to benefit from these new studies. Which will be a bit of a relief because there are many diseases, not just Zika, not just dengue, there are many diseases that do we do not have a cure for  and it would be very good if this new generation is the beneficiary so that so many people don’t continue to die.” |
| **COVID-19-related benefit sharing** |  |  |
| (C22-DAR, Colombia,  less than 25 years old,  male, participant) | “Digamos por ejemplo que tienen el desarrollo de la vacuna del zika, y esa vacuna sirve para salvar a alguien… esto…digamos que sea yo…yo lo voy a necesitar en caso de que este infectado, yo lo voy a necesitar para obtenerlas…y ahí… como voy a hacer, digamos… yo aporté mis muestras para obtenerla… entonces como voy a hacer, me va a valer un platal (la vacuna) o bueno, va a ser difícil obtenerla, no es de fácil alcance para algunas personas, y todo esto porque ese descubrimiento, ahora…. como tal es de un grupo, entonces ellos se ven beneficiados económicamente, y pues… no les interesa… entonces no les interesaría beneficiar a la sociedad, que es como yo creo que debería ser, y que fue la razón….como te dije en un principio, para eso di la muestra. | "Let's say for example that they have the development of the Zika vaccine, and that vaccine is used to save someone... this... let's say it is me... I am going to need it in case I am infected, I am going to need it to obtain it... and then... how am I going to do, let's say... I contributed my samples to obtain it... so how am I going to do it? how am I going to do, let's say... I contributed my samples to obtain it... then how am I going to do, it is going to be worth a lot (the vaccine) or well, it is going to be difficult to obtain it, it is not easy to reach for some people, and all this because this discovery, now.... as such it belongs to a group, so they benefit economically, and well... they are not interested... so they are not interested in benefiting society, which is how I think it should be, and that was the reason .... as I told you at the beginning, that is why I gave the sample." |
| (C18-NAP, Colombia, 25-34 years old, female, participant) | “ Y pues como ahora… que tal yo dando la sangre para la vacuna del COVID y que no me pueda vacunar” | "(...) how about me giving blood for the COVID vaccine and not being able to get vaccinated?." |
| **Data and sample sharing with the pharmaceutical industry** |  |  |
| (N14-JNP, Nicaragua, 35-45 years old, female, respondent's mother) | “Pienso que es muy bueno porque así se desarrollarían más medicinas adecuadas a la enfermedad” | "I think it's very good because that way more medicines suitable for the disease would be developed." |
| (N13-MNRR, Nicaragua, 35-45 years old, female, respondent's parent) | “[Son] avances para ellos, pero que están comprando algo que mi hija y yo estamos aportando sin ningún costo solo por ayudar a la investigación y que ellos pueden venir y comprar sin  que nosotros sepamos y ellos estarían obteniendo un beneficio económico, con algo que nosotros estamos donando sin obtener nada a cambio solo por ayudar.” | “[They are] advances for them, but that they are buying something that my daughter and I are contributing at no cost just to help the investigation and that they can come and buy without our knowledge and obtain an economic benefit, with something that we we are donating without getting anything in return just to help.” |
| (N14-JNP, Nicaragua, 35-45 years old, female, respondent's parent) | “Ahí si no sería tan feliz porque cuando son compartidas con los laboratorios y traen [como fuera del país] medicamento, inyecciones, pastillas, etc. Y son comerciales, los venden y a veces muy caros, que el ser humano no puede pagar. Los de escasos recursos no pueden pagar, solo los grandes millonarios que tienen billete, nosotros los pobres no” | “There, I would not be so happy because when they are shared with laboratories and they bring [outside of the country] medicine, injections, pills, etc. And they are commercial, they sell them and sometimes they are very expensive, so that human beings cannot pay. Those with limited resources cannot pay, only the great millionaires that have enough money, not us poor people.” |
| (P6-ASU, Colombia, more than 45 years old, female, respondent's parent) | “Ese es el problema, que uno no sabe si es para bien… o sea que sacaran… porque es que solo le sacan sangre al niño y es un poquito no ve? Entonces, no se…, pero de todas formas si es para que la gente se beneficiara, ¿si ve? Pues si… Que si era… que si es para sacar alguna droga, alguna vacuna, pues sí, me gustaría, que la otra gente se beneficiara también. Pues yo la verdad confío en que ustedes hagan las cosas bien” | "That's the problem, you don't know if it is for [a good purpose]...but in any case, if it's for the benefit of the people…if it is to get some drug, some vaccine, well yes, I would like other people to benefit as well. Well, in truth, I have faith that you will do things well.” |
| (C22-DAR, Colombia, less than 25 years old,  male, participant) | “Digamos que… yo la voy a necesitar (la vacuna) en caso de que esté infectado (...) y ahí… ¿Cómo voy a hacer? digamos… yo aporté mis muestras para obtenerla (la vacuna) entonces ¿cómo voy a hacer? me va a valer un platal o bueno, va a ser difícil obtenerla. No es de fácil alcance para algunas personas, y todo esto porque ese descubrimiento, ahora, es de un grupo (los patrocinadores), entonces ellos se ven beneficiados económicamente, y pues… entonces no les interesaría beneficiar a la sociedad, que es como yo creo que debería ser, y que fue la razón, como te dije en un principio, para eso di la muestra. Creo que la ciencia, las investigaciones deben ser para beneficio de todos y no se tiene que ver como un beneficio económico…. como tal… porque estamos hablando de salud, no estamos para comercializar, entonces eso es lo que a mí me gustaría”. | “Let's say I'm going to need [the vaccine] in case I'm infected and then what am I going to do?  Let's say, I contributed my samples to obtain [the vaccine] so, what am I going to do?  It's going to cost a ton of money or, well, it's going to be difficult to get.  It won't be easily accessible to some people, and all this because that discovery now belongs to a group [the sponsors], so they benefit financially, and well then they would not be interested in benefiting society, which is how I think it should be. And that was the reason, as I told you at the beginning, that's why I gave the sample.  I believe that science and research should be for the benefit of everyone and should not be seen as an economic benefit. As such, because we are talking about health, we are not here to commercialise, so that is what I would like.” |
| **Trust in the research team** |  |  |
| (C14-DMM, Colombia, 35-45 years old, female, respondent's parent). | “Pues yo confío, primero en la universidad XX… y se que no le van a entregar (muestras, datos) a cualquier grupo de investigación o cualquier laboratorio porque sí, debe  tener un proyecto, un estudio, no es todo de la noche a la mañana. Aino que todo es como bien estructurado ¿no? Son profesionales y muy éticos” | “Well, I trust, first in the XX university… and I know that they are not going to deliver (samples, data) to whichever research group or any laboratory because yes, they must have a project, a study, it is not all [something that happens] overnight. Rather everything is well structured, right? They are professional and very ethical.” |
| (C18-NAP, Colombia, 25-34 years old, female, respondent's parent) | “Ya participé en ese estudio del dengue…y me fue bien porque mantuvieron ahí periódicamente. Me estuvieron llamando…preguntándole a uno que como se encuentra y todo eso. Entonces ya uno lo conoció anteriormente. Entonces me imagino que esta nueva etapa con el zika sea igual.” | “I already participated in that dengue study… and it went well for me because [the doctors] stayed there off and on. They were calling me. They were calling people asking how they are doing and all that…So I imagine that this new stage with Zika will be the same.” |
| (C20-EM, Colombia, more than 45 years old, female, respondent's parent) | “Hasta ahora se han portado muy bien, vienen y me visitan, están pendientes del niño. Entonces debe ser que están haciendo algo bueno los de la universidad XX” | "Until now they have behaved very well, they come and visit me, they look out for the child. So it must be that the people from XX university are doing something good." |
| **Overall perception of the cognitive interview study** |  |  |
| (C17-MSF, Colombia, more than 45 years old, female, respondent's parent) | “No tenía ni la menor idea de que para hacer un estudio pedían tanto permiso, como tomar las muestras, para guardarlas, para usarlas en el futuro. No tenía la más mínima idea porque yo pensaba que eso lo hacían sin permiso de uno. El hecho de que ustedes me estén diciendo a mí que lo va hace para esto o para lo otro, para analizar el genoma, para analizar el ADN, me da confianza....” | “I had no idea that to carry out a study they asked for so many permissions, such as taking the samples, to store them, to use them in the future. I had no idea because I thought that they did it without getting your permission. The fact that you are telling me that [the investigator] is going to do it for this or that, to analyze the genome, to analyze the DNA, gives me confidence…” |
| (C18-NAP, Colombia, 25-34 years old, female, participant’s mother) | “He aprendido mucho. Muchas cosas yo nunca las había escuchado y sigo pensando en toda esa información, porque todo eso le deberían decir a uno cuando le van a sacar la sangre para esos estudios. Yo nunca había escuchado todas esas cosas.” | “I have learned a lot. Many things I had never heard before and I keep thinking about all that information, because they should tell you when they are going to take your blood for those studies. I had never heard all those things.” |
| (N15-LMMU,  Nicaragua, 35-45 years old, female, respondent's parent) | “Pero es que mire, en un momento a veces pasa esto, uno firma y uno no se da ni cuenta, hasta preso te pueden llevar, porque uno no sabe en el momento del apuro en el que está, entonces eso nos queda de experiencia para aprender y fijarnos que es lo que estamos haciendo.” | "But look, in the moment, sometimes, it happens that you sign and you don't even realize it, they can even take you to prison, because you don't know at the moment when they are so rushed, so this becomes an experience we can learn from, so that we pay attention to what we are doing." |
| **Cross group differences in the understanding and acceptance of broad consent for future use of data and samples** |  |  |
| (C24-MSB, Colombia, less than 25 years old, male, participant) | Yo realmente no tendría que reclamar porque lo único que estoy haciendo es dando un aporte con una muestra, ayudándolos (...) ese es mi aporte…Pero los investigadores tampoco podrían llegar a esos estudios, a esas respuestas que quieren llegar, sino es con mi ayuda y la de muchos más, ¿ves? | “I really would not have to complain because because the only thing I'm doing is giving a contribution with a sample, helping them (...) that's my contribution....But the researchers wouldn't be able to get to those studies either, to those answers that they want to get to, without my help and that of many others, you see?” |
| (C22-DAR, Colombia, less than 25 years old,  male, participant) | “Como yo le dije, yo soy técnico en administración y estoy estudiando administración… muchas veces las empresas o sobre todo los grupos, grupos grandes, y en la investigación también tienen que haber esos grupos, lo que quieren hacer es como aprovecharse o en caso tal ser los primeros de que lleguen a una respuesta que todavía no tiene nadie… y al momento de tener eso, ¿si digamos hay varios grupos que tienen las mismas respuestas, pues ellos mismos se unen para qué? Para sacar un beneficio económico, entonces alzan los precios. | “As I told you, I am an administrator and I am studying administration…many times companies or especially groups, large groups, and in research there also have to be those groups, what they want to do is how to take advantage or in such a case, be the first to arrive at an answer that no one has yet… and at the moment of having that, if, let's say, there are several groups that have the same answers, then why do they unite themselves? To make an economic profit, then they raise prices.” |
| (C15-CCT, Colombia, less than 25 years old,  male, participant) | “....cuando yo participe en el estudio… yo no recibí toda esta información entonces usted me pone a pensar en que mi mama por ejemplo no tiene ni idea de estas cosas, entonces ella solo dijo bueno. Pero porque no sabe nada si ve?” | “...When I participated in the study… I did not receive all this information. So you make me think that my mom, for example, has no idea about these things. So she just said “okay,” but because she doesn't know anything, you understand?” |
| **Parental consent for sharing data or samples from their children** |  |  |
| (N6-MPHM, Nicaragua-35-45 years old, female, respondent's parent) | “siento como que le ayudamos a otras personas, porque tal vez hay personas que no permiten que le tomen alguna muestra de sangre para hacer investigaciones. En este caso siempre tomo el consentimiento con ella, si ella decide sacarse sangre porque ella ya es una señorita y también tiene su voz y su voto en decir sí o no. Entonces siento que nosotros estamos dando un granito de arena, ese apoyo por parte de ella para que se procedan hacer investigaciones, no importando los datos de ella ni mis datos personales” | “I feel like we help other people, because maybe there are people who do not allow them to take their blood sample for research. In this case, I always take the consent with her [my daughter] if she decides to give the blood because she is already a lady and she also has her voice and her vote in saying yes or no. So I feel that we are giving a grain of sand, that support on her part so that the investigations can proceed, regardless of her data or my personal data.” |
| (N17-JGG, Nicaragua, more than 45 years old, male, respondent's parent) | “Desde que hicieron el estudio del Dengue yo le consulté a ella, a mis hijos siempre le hemos dado un poquito, digamos que ellos tomen decisiones desde pequeños. Entonces yo estoy haciendo esto no porque yo lo decidí sino que también ella. La tomo en cuenta, en ese sentido yo busco como ella vaya desarrollando un poquito más y ella no está renuente hacer este tipo de cosas” | "From the time [they started] the dengue study, I consulted [my daughter], we have always given my children a little bit [of responsibility], we have said that they make decisions from an early age. So I am doing this not because I decided, but because she also [decided].." |
| **Recommendations for future research participants** |  |  |
| (P5, Colombia, 25-34 years old, male, participant) | “Que no hay que pensar en lo personal sino en lo grupal, que hay que ayudarnos unos a otros, apoyarnos como hermanos y darle la oportunidad a un futuro enfermo por decirlo así. Que tenga una medicina que pueda ser salvaguardado más rápidamente que en un pasado.” | “you shouldn't think about the individual, but rather about the group, that we must help each other, support each other as brothers and give the opportunity to a future patient, so to speak. So that they have a medicine that can be safeguarded more quickly than in the past.” |
| (C16-YMA, Colombia, less than 25 years old, female, respondent's parent) | “Que primero lean bien y que comprendan la información que le están dando los investigadores para que sea todo comprendido y responder de la mejor manera.” | "That first [the participants] read [the ICF] well and that they understand the information that the researchers are giving them so that everything is understood and they respond in the best way." |
| **Recommendations to researchers on how to improve broad consent** |  |  |
| (P3-JS, Colombia, 35-45 years old, female, respondent's parent) | “Yo les diría que me parece muy bien todo lo que hacen y que, al igual que ustedes los investigadores, nosotros somos personas dispuestas a colaborar para que trabajemos en conjunto. Pero que se expliquen bien las cosas… para que uno les pueda creer siempre” | “I would tell them that everything they do seems very good to me and that, just like you researchers, we are people willing to collaborate so that we work together. But that things should be explained well...so that you can always believe them.” |
| (N17-JGG, Nicaragua, more than 45 years old, male,respondent's parent) | “Bueno, yo no me explico, vuelvo y le repito disculpe la palabra como nicaragüense, me chivea eso. Ja, ja, ja! Que me diga que van a mantenerlo en secreto, como que a mi hija después de ese estudio me la van a sacar, me la van a venir a secuestrar eso es lo que me da a entender” | "Well, I don't explain myself, I come back and I repeat, excuse me for the word as a Nicaraguan, that chivetes me.  Ha, ha, ha, that you tell me that they are going to keep it a secret, like that my daughter after that study is going to be take her out, they're going to come and kidnap her, that's what makes me understand.” |
| (C18-NAP, Colombia, 25-34 years old, female, participant) | “lo que no entiendo es para que piden tantas autorizaciones. Parece que como que es delicado eso de las muestras de sangre… sino porque piden tanta autorización y para qué dicen tantas cosas” | “What I don't understand is why they ask for so many authorizations. It seems that blood samples are delicate… but because they ask for so much authorization and why they say so many things.” |
