# Supplementary material for: "How about me giving blood for the COVID vaccine and not being able to get vaccinated?" A cognitive interview study on understanding of and agreement with broad consent for future use of data and samples in Colombia and Nicaragua
Source: PLOS Glob Public Health. 2023 May 17;3(5):e0001253. doi: 10.1371/journal.pgph.0001253 (PMC10191364; doi:10.1371/journal.pgph.0001253)
Supplement: S4 Table — (DOCX) [file pgph.0001253.s004.docx]

**S4 Table. Confusing phrases in the University of Berkeley informed consent template and suggested language**

| **Original language - Spanish** | **Original language - English** | **Interpretation** | **Suggested language - Spanish** | **Suggested language - English** |
| --- | --- | --- | --- | --- |
| *Tenemos previsto* conservar y compartir la información | We plan to keep the information and samples collected from your child in case we or other researchers want to use it later for other studies. | Difficulty understanding what is meant by this process and what information will be preserved. | Ahora, queremos pedir su autorización / permiso para guardar un poquito de esa sangre y algunas de sus respuestas | Now, we would like to ask for your authorization/permission to keep a little bit of that blood and some of your answers. |
| …y las muestras recogidas … |  | The word "samples" was not associated with a human-derived biospecimen | Se sugiere acompañar la palabra “muestra” con el tejido a recolectar, en este caso, sangre. Podria ser saliva, orina, etc. | Accompany the word "sample" with the tissue to be collected, in this case, blood. It could be saliva, urine, etc. |
| …la información o las muestras recogidas podrían ser utilizadas por nuestro equipo para futuros estudios de investigación o distribuidas a otros investigadores para futuros estudios de investigación, sin su consentimiento informado adicional… | After we remove any identifying information, the information or biospecimens collected from your child could be used by our team for future research studies or distributed to other investigators for future research studies without additional informed consent from your child or from yourself. | Se entiende que usar la información o las muestras en “futuros estudios de investigación”, es usarlas en cualquier tipo de estudio. La frase “consentimiento informado” no se interpreta como un permiso para… | …la información o las muestras de sangre recogidas de su hijo/a podrían ser utilizadas por nuestro equipo o por otros investigadores para futuros estudios vinculados a la salud, sin pedirle a usted, nuevamente su permiso… | ...information or blood samples collected from your child may be used by our team or other researchers for future health-related studies without your permission, again without asking you... |
| …tendrá que proporcionar información sobre cómo se mantendrá la confidencialidad de los datos o las muestras… | Any study that uses your child’s data or samples would have to provide documentation for how the confidentiality of your child’s data or samples will be maintained and how the future research could benefit your community or public health more generally. | The concept of confidentiality was difficult to understand. | …tendrá que decirnos dónde y cómo guardará de manera segura la información de sus datos y de sus muestras… | ...you will need to tell us where and how you will securely store your data and sample information... |
| …usted no participará de ningún valor comercial o beneficio derivado del uso de sus muestras… | You will not share in any commercial value or profit derived from the use of your child’s biospecimens and/or information obtained from them. | Commercial value was not well understood. | …usted no recibirá ningún beneficio, ni económico, ni de ningún otro tipo… | ...you will not receive any benefit, financial or otherwise... |
| …incluirá la secuenciación del ADN o ARN del genoma completo… | The research will include whole genome DNA or RNA sequencing. | Understood as paternity detection | Se propone explicar con detalle el significado de “secuenciación” “ADN” y “genoma” | It is proposed to explain in detail the meaning of "sequencing" "DNA" and "genome". |
| Cualquier muestra adicional será almacenada en un biobanco de Colombia. | Any extra samples will be stored in a biobank | No se entiende el concepto de “muestra adicional”. El término “biobanco” es más claro, en la medida que se explique qué se quiere guardar en ese lugar. | La sangre que quede de sobra *será almacenada en un biobanco* de Colombia | Leftover blood will be stored in a biobank in Colombia. |
| …dicho uso podría dar lugar a invenciones y descubrimientos que podrían convertirse en la base de nuevos productos comerciales, pruebas de diagnóstico o agentes terapéuticos… | The DNA samples and information will be used for research, and such use could possibly result in inventions and discoveries that could become the basis for new commercial products, diagnostic tests, or therapeutic agents. | Las palabras “producto comercial” se interpretan como cualquier producto que salga a la venta, como shampoo, jabón, etc. Las palabras “pruebas diagnósticas” y “agentes terapéuticos” no se comprenden | …dicho uso podría convertirse en la base para aportar al desarrollo de nuevos productos de uso comercial o no comercial. Para nuevas pruebas para detectar el zika o para agentes terapéuticos como medicamentos. | ...such use could become the basis for contributing to the development of new products for commercial or non-commercial use. For new tests to detect Zika or for therapeutic agents such as drugs. |
| *la información de identificación*, como el nombre, la dirección y la fecha de nacimiento de su hijo, se eliminará del registro de su hijo o del registro asociado a su muestra… | Identifying information, like your child’s name, address, and birthdate, will be removed from your child’s record or from the record associated with their biospecimen before we share their data or biospecimen with any other group. | No se asocia el concepto de “eliminar la información personal” con la obligación del investigador de proteger la identidad del participante y por tanto de anonimizar la información. | Vamos a proteger su información *de identificación*, como el nombre, la dirección y la fecha de nacimiento. Eso se debe hacer de manera obligatoria en todas las investigaciones, antes de compartir sus datos o su muestra de sangre con cualquier otro grupo de investigación… | We will protect your identifying information, such as name, address and date of birth. This must be done on a mandatory basis in all research before sharing your data or your blood sample with any other research group... |
| *… los resultados de la* *investigación* que sean clínicamente relevantes, incluyendo los resultados individuales de la investigación, no serán revelados a los sujetos participantes | Clinically relevant research results, including individual research results, will not be disclosed to subjects. | Los resultados clínicamente relevantes son percibidos y esperados como un beneficio individual. Podría estar asociado i) al contexto de escasez ii) a dificultades para acceder a los servicios de salud iii) a la falta de comprensión en relación con el propósito de una investigación y la diferencia entre atención médica para diagnóstico. | Quiero recordarle que el objetivo de una investigación es obtener más conocimiento sobre un tema específico, no es hacer un diagnóstico médico. Esta investigación la haremos utilizando muestras de sangre. Otras investigaciones podrían requerir orina, saliva, etc.. Esas muestras son analizadas en el laboratorio pero los resultados individuales no serán comunicados | I want to remind you that the objective of an investigation is to gain more knowledge about a specific topic, not to make a medical diagnosis. This research will be done using blood samples. Other investigations may require urine, saliva, etc. Those samples are analyzed in the laboratory but the individual results will not be communicated. |
