# Supplementary material for: "How about me giving blood for the COVID vaccine and not being able to get vaccinated?" A cognitive interview study on understanding of and agreement with broad consent for future use of data and samples in Colombia and Nicaragua
Source: PLOS Glob Public Health. 2023 May 17;3(5):e0001253. doi: 10.1371/journal.pgph.0001253 (PMC10191364; doi:10.1371/journal.pgph.0001253)
Supplement: S1 Text — (DOCX) [file pgph.0001253.s005.docx]

**Supplementary Text S1. Interview guide, including demographic survey and cogntive interview, that evaluates understanding of language from the Spanish translation of the University of California at Berkeley Spanish-language template informed consent for biomedical studies**

1. **GUÍA DE RECLUTAMIENTO**

Nos estamos poniendo en contacto con usted porque su hijo / usted participa en el estudio XX. ¿Por favor me confirma si estoy en lo correcto? Dígame por favor ¿hace cuánto tiempo aceptó participar en ese estudio? / ¿Hace cuánto tiempo acepto que su hijo participara en ese estudio?

Ahora le estamos invitando a participar en un nuevo estudio, que realizan investigadores de la Universidad Industrial de Santander, el Instituto de Ciencias Sostenibles y la Universidad de Heidelberg y estamos pidiendo la participación de 24 personas.

En este estudio queremos explorar cómo los participantes de las investigaciones, como usted, entienden las palabras que usan los investigadores para describir el uso de muestras y datos. Si logramos comprender la manera en que usted entiende esas palabras, podremos ayudar a los investigadores a hacer un mejor trabajo cuando explican estos temas a las personas que participan en un estudio de investigación.

La conversación que queremos tener con usted, dura aproximadamente 2 horas y lo haremos en el momento que le resulte más cómodo. Si está de acuerdo, lo hacemos el mismo día descansando 15 a 20 minutos cada hora. Sino, podemos programar 2 encuentros. Lo haremos como a usted le parezca mejor.

En la primera parte de la conversación, le haremos algunas preguntas relacionadas con su edad, sexo y trabajo; y le leeremos algunas frases sobre el uso de muestras y datos y le pediremos que nos diga lo que le viene a la mente cuando escucha esas frases y cómo las entiende. Durante la segunda parte de la conversación, haremos una encuesta que se llama Banks-SP.

Si usted lo permite vamos a grabar sus respuestas y luego las vamos a transcribir. Durante la transcripción borraremos cualquier información que permita identificarlo. Una vez tengamos listo el texto, borraremos el audio grabado.

En agradecimiento por su participación y para compensar el tiempo que le dedicará a esta conversación, le entregaremos $ 30.000.oo

Cuénteme qué opina sobre lo que le he dicho ¿le gustaría participar?

1. **PROCESO DE CONSENTIMIENTO INFORMADO**

Ver texto adjunto al protocolo enviado a los CEI de Colombia y Nicaragua

1. **CUESTIONARIO DEMOGRAFICO**

Señor (a) gracias por participar en este proyecto de investigación. En días pasados conversé (conversaron) con usted, sobre lo que vamos a hacer durante esta conversación. ¿Todo está claro? ¿O prefiere que recordemos algunos aspectos? / ¿O tiene alguna preocupación al respecto de este estudio?

Bien, una vez resueltas las inquietudes vamos a empezar:

Con respecto a quien responde el cuestionario:

1. ¿Es usted participante o usted fue quien dio autorización para la participación de su hijo/a en el estudio? ¿Me podría decir cuál es el parentesco de usted con el niño-a?
2. Vive en el área rural o urbana (de Piedecuesta)
3. ¿Cuántos años cumplidos tiene?
4. ¿Se identifica como hombre o mujer?
5. ¿Cuántos años de educación formal ha completado?
6. ¿Cuál es su ocupación? / ¿A qué se dedica?
7. En su casa hablan español. ¿cierto?
8. **GUIA DE ENTREVISTA COGNITIVA**

***Instrucciones para el entrevistador***

*Explique al participante en qué consiste la entrevista cognitiva. Tenga a mano el resumen de un estudio hipotético que servirá como base de contexto para la entrevista. Para la segunda parte, lea cada sección del guion y reléala para aplicar las técnicas cognitivas de reporte verbal, como el parafraseo y las pruebas de especificación de la siguiente manera:*

- *¿Podría decirme con sus palabras lo que acabo de leer?*
- *¿Podría decirme, qué significa para usted la palabra XXX (o la frase XXX)? ¿Podría darme ejemplos por favor?*
- *Usted dijo “depende”. ¿Podría decirme, depende de qué? (Por ejemplo).*

1. **ENTREVISTA COGNITIVA**

***Introducción a la entrevista***

Señor (a) XXX., vamos a hablar de algunos temas que generalmente se mencionan, cuando se contacta a las personas para que participen en estudios de investigación que incluyen un consentimiento amplio. Como usted ha participado en ese tipo de estudios es muy probable que algunas frases ya le sean familiares. Si no, no hay problema, poco a poco las vamos a recordar.

Señor (a) XXX., durante esta entrevista, vamos a hacer un ejercicio que se llama “parafraseo para la comprensión”. Para hacerlo vamos “a hacer de cuenta” que lo / la estoy invitando a participar de un estudio y al final le pediré que me diga “con sus palabras” qué entiende por algunas frases o palabras relacionadas con el uso de muestras y datos para estudios futuros.

Recuerde señor (a) XXX., este ejercicio lo vamos a hacer sobre un estudio que no es real, pero que se lo voy a narrar como si lo fuera.

¿Está listo / a? Recuerde, ahora yo soy un investigador y usted es un /a posible candidato/ a para participar en un estudio. ¿Empezamos con la primera parte?

1. *Introducir el lenguaje del consentimiento informado amplio*

Señor (a) XXX., muy buena tarde. Lo contacto para invitarlo a participar en el estudio “Grado de afectación del ZIKA en la población infantil y adulta de Colombia”. *(Para contextualizar al participante, se le hará un corto resumen sobre ¿Qué es el zika? ¿Quiénes pueden participar? ¿Objetivo del estudio? ¿Procedimientos a realizar durante el estudio? ¿Riesgos y beneficios?)*

Señor (a) XXX., ¿tiene alguna pregunta en relación con el estudio? Muy bien, una vez aclaradas sus inquietudes, en adelante, le voy a solicitar que nos permita compartir las muestras de sangre que tomaremos en el estudio.

Excelente trabajo Señor (a) XXX., ahora vamos a iniciar la segunda parte que está relacionada con el uso de las muestras en estudios futuros. Recuerde que estamos “haciendo de cuenta” que lo estoy invitando a participar en el estudio “Grado de afectación del zika en la población infantil y adulta de Colombia”. Ya le dije de qué se trata el estudio; ahora, vamos a revisar algunos temas relacionados con el uso futuro de muestras. ¿Continuamos?

Señor (a) XXX., quiero comentarle que para este estudio del zika, “*tenemos previsto…”*

**Uso futuro (información para el investigador, no para el participante)**

*Tenemos previsto* conservar y compartir la información y las muestras recogidas de su hijo en caso de que nosotros u otros investigadores queramos utilizarlas posteriormente para otros estudios.

Voy a repetir XXX: *tenemos previsto*…

*Indagar*

- ¿Qué le quise decir con eso? dígame con sus palabras lo que le acabo de decir.
- ¿En qué pensó usted cuando escuchó esto?
- Usted mencionó XXXX, cuénteme más sobre eso.
- Cuando digo que vamos a conservar y compartir “muestras,” ¿qué significa para usted? ¿qué entiende por “muestras biológicas”?
- ¿Qué cree que pasará con la sangre de su hijo que fue recogida?

Excelente, vamos con otra frase. El mismo ejercicio pero con otra frase:

Señor (a) XXX., quiero comentarle que para este estudio del zika, *la información de identificación*, como el nombre, la dirección y la fecha de nacimiento de su hijo, se eliminará del registro de su hijo o del registro asociado a su muestra antes de que compartamos sus datos o su muestra con cualquier otro grupo de investigación.

Le vuelvo a leer… *la información de identificación*…

- ¿Qué le quise decir con eso? dígame con sus palabras lo que le acabo de decir.
- ¿Qué cree que significa, que la información de identificación de su hijo se eliminara del registro asociado a su muestra?
- Usted mencionó a XX, cuénteme más sobre eso.

Excelente, vamos con otra frase. El mismo ejercicio pero con otra frase:

Señor (a) XXX., quiero comentarle que en este estudio del zika, *después de eliminar cualquier información de identificación*, la información o las muestras recogidas de su hijo podrían ser utilizadas por nuestro equipo o por otros investigadores para futuros estudios, sin el consentimiento informado adicional de su hijo o de usted mismo.

Le vuelvo a leer… *después de eliminar cualquier información de identificación…*

- ¿Qué le quise decir con eso? dígame con sus palabras lo que le acabo de decir
- ¿Qué cree que significa, que nuestro equipo podría usar la información o la sangre recolectado de su hijo para otros estudios o compartirlos con otros equipos de investigación?
- Y ¿Cómo se siente usted cuando escucha eso?
- Usted dice XXXXX…. explícame un poco más acerca de esto.

Muy bien señor (a) XXX., ahora, si le digo que pensamos compartir las respuestas o las muestras, como la sangre, con equipos de investigación de otros países para, por ejemplo, desarrollar nuevas vacunas para el zika…

- ¿Qué le quise decir con eso? dígame con sus palabras lo que le acabo de decir
- Y ¿Cómo se siente usted cuando escucha eso?
- Usted dice XXXXX…. explícame un poco más acerca de esto.

Excelente, vamos con otra frase:

Señor (a) XXX., quiero comentarle que *cualquier estudio que utilice los datos o las muestras* de su hijo recolectadas en este estudio del zika, tendrá que proporcionar información sobre cómo se mantendrá la confidencialidad de los datos o las muestras de su hijo

Le vuelvo a leer… *cualquier estudio que utilice los datos o las muestras…*

- ¿Qué le quise decir con eso? dígame con sus palabras lo que le acabo de decir
- ¿Qué significa mantener la confidencialidad?
- Y ¿Cómo se siente usted al permitir que su hijo participe en ese estudio?
- Usted dice XXXXX…. explícame un poco más acerca de esto.

Señor (a) XXX., quiero comentarle que *cualquier estudio que utilice los datos o las muestras* de su hijo recolectadas en este estudio del zika, tendrá que proporcionar información sobre cómo se mantendrá la confidencialidad de los datos o las muestras de su hijo y cómo la futura investigación podría beneficiar a su comunidad o a la salud pública en general.

Le vuelvo a leer… *cualquier estudio que utilice los datos o las muestras…*

¿Qué le quise decir con eso? dígame con sus palabras lo que le acabo de decir

¿Cómo entiende usted los beneficios que puede recibir la comunidad? ¿Qué significa beneficios para la salud pública?

¿Cómo se siente de participar / usted o su hijo/ en este estudio?

Vamos nuevamente con otras frases:

**Cláusula Moore (información para el investigador, no para el participante)**

Señor (a) XXX., quiero comentarle que *las muestras recogidas de su hijo para* este estudio del zika, y/o la información obtenida de esas muestras, pueden utilizarse en esta investigación o en otras investigaciones, y compartirse con otras organizaciones.

Le vuelvo a leer… *las muestras recogidas de su hijo para el…*

- ¿Qué le quise decir con eso? dígame con sus palabras lo que le acabo de decir
- ¿Qué significa, que la información obtenida de esas muestras, compartirse con otras organizaciones?
- Y ¿Cómo se siente usted al permitir que su hijo participe en ese estudio?
- Usted dice XXXXX…. explícame un poco más acerca de esto.

Señor (a) XXX., quiero comentarle que las muestras recogidas de su hijo (o suyas) para este estudio del zika, y/o la información obtenida de esas muestras, pueden compartirse con otras organizaciones y usted no participará de ningún valor comercial o beneficio derivado del uso de las muestras de su hijo y/o de la información obtenida de ellos.

Le vuelvo a leer… *las muestras recogidas de su hijo para el…*

- ¿Qué le quise decir con eso? dígame con sus palabras lo que le acabo de decir
- ¿Qué significa, que usted no participará de ningún valor comercial o beneficio derivado del uso de las muestras de su hijo?
- Y ¿Cómo se siente usted al permitir que su hijo participe en ese estudio?
- Usted dice XXXXX…. explícame un poco más acerca de esto.

Señor (a) XXX., quiero comentarle que este estudio del zika, incluirá la secuenciación del ADN o ARN del genoma completo.

Le vuelvo a leer… *quiero comentarle que este estudio del zika…*

- ¿Qué le quise decir con eso? dígame con sus palabras lo que le acabo de decir
- ¿Qué significa, incluir la secuenciación del ADN o ARN del genoma?
- Y ¿Cómo se siente usted al permitir que su hijo participe en ese estudio?
- Usted dice XXXXX…. explícame un poco más acerca de esto.
- Para usted, ¿qué quiere decir genoma?

Señor (a) XXX., quiero comentarle que *los resultados de* este estudio del zika que sean clínicamente relevantes, incluyendo los resultados individuales de la investigación, no serán revelados a los sujetos participantes

Le vuelvo a leer… *los resultados de la* *investigación…*

- ¿Qué le quise decir con eso? dígame con sus palabras lo que le acabo de decir
- ¿Qué entiende por resultados clínicamente relevantes? Por favor, dígame lo mismo con sus propias palabras
- ¿Qué entiende por resultados individuales de la investigación?
- Usted dice XXXXX…. explícame un poco más acerca de esto.

Excelente señora XXX. Ahora voy a leer nuevamente todo el texto pero, esta vez, al final usted va a tomar una decisión con base en la información. ¿Listo?

Las muestras recogidas de su hijo para este estudio y/o la información obtenida de esas muestras, pueden utilizarse en esta investigación o en otras investigaciones, y compartirse con otras organizaciones. Usted no participará de ningún valor comercial o beneficio derivado del uso de las muestras de su hijo y/o de la información obtenida de ellos. La investigación incluirá la secuenciación del ADN o ARN del genoma completo. Los resultados de la investigación clínicamente relevantes, incluyendo los resultados individuales de la investigación, no serán revelados a los sujetos participantes. Calculamos que nuestro estudio tardará 5 años o más en completarse.

Por favor, elija una de las siguientes opciones:

1. Usted permite que las muestras de su hijo se utilicen para este estudio y se almacenen para futuros estudios o
2. Usted permite que las muestras de su hijo se utilicen para este estudio PERO solicita que se destruyan después de la finalización de este estudio y no se utilicen en futuros estudios

*Le vuelvo a leer… ¿desea que lea todo el párrafo o únicamente las preguntas? Bien, antes de conocer su respuesta, me gustaría saber lo siguiente:*

- ¿Qué le quise decir con cada pregunta? ¿Lo recuerda? ¿sobre qué tenía que elegir? ¿Cuáles eran las opciones?

*Explicar las opciones si no fueron claras para el participante*…

- Ahora, si usted estuviera participando nuevamente en ese estudio, y tuviera que elegir entre esas dos opciones ¿Cual escogería usted?

- ¿Por qué? Cuéntame más acerca de esto.

Y si usted dijo que NO DA PERMISO, ¿hasta cuándo piensa que se guardaran las muestras?

Muy bien señor XXX continuemos con el ejercicio. Lo está haciendo muy bien. Es genial hacer este tipo de trabajo con personas como usted.

**Datos genéticos (información para el investigador, no para el participante)**

Vamos con esta frase:

Señor (a) XXX., quiero comentarle que para este estudio del zika *nos gustaría contar con un permiso* adicional para almacenar las muestras de sangre y el ADN de su hijo para futuros estudios genéticos. Los estudios genéticos examinan la información genética que se transmite de padres a hijos.

Le vuelvo a leer… quiero comentarle que para este estudio del zika *nos gustaría contar con un permiso…*

- ¿Qué le quise decir con eso? Dígame con sus palabras lo que usted entendió
- Usted mencionó XX, cuénteme más sobre eso.
- Usted menciono XXXX cuénteme más acerca de esto.

Señor (a) XXX., quiero comentarle que para este estudio del zika, en concreto, buscaremos genes que puedan hacer que las personas tengan más o menos probabilidades de desarrollar enfermedades graves causadas por infecciones de virus como el zika, Chikunguña y otros genes relacionados con el estado de salud.

- ¿Recuerda que en algunas preguntas anteriores hablamos sobre datos genéticos? Entonces quiero saber, como está ahora su comprensión sobre ese tema, sobre datos genéticos
- ¿Qué significa que vamos a buscar genes?

Señor (a) XXX., *se espera que esta investigación* sobre el zika *dure muchos años.* Si usted decide no darnos su permiso, puede seguir participando en el estudio y esto no afectará a la atención médica que recibe su hijo.

Voy a leer nuevamente: *se espera que esta investigación* sobre el zika *dure muchos años…*

- ¿Qué le quise decir con eso? Dígame con sus palabras lo que usted entendió
- Usted mencionó a XX, cuénteme más sobre eso.
- ¿Cuántos años cree que durará el estudio?
- Para usted qué significa confidencialidad de la información.

Señor (a) XXX., *las muestras de ADN y la información clínica se enviarán, sin el nombre* de su hijo, a uno de nuestros socios de investigación y podrán enviarse a otros grupos de investigación que colaboren con nuestro laboratorio en el futuro.

Voy a leer nuevamente: *las muestras de ADN y la información clínica se enviarán, sin el nombre…*

- ¿Qué le quise decir con eso? Dígame con sus palabras lo que usted entendió
- Usted mencionó a XX, cuénteme más sobre eso.
- ¿Qué significa para usted, muestras de ADN?
- ¿Qué piensa usted sobre la forma en que manejaremos las muestras de sangre y la información genética de su hijo?
- ¿Cuáles son sus preocupaciones?

Señor (a) XXX., cualquier muestra adicional *será almacenada en un biobanco* de Colombia.

- ¿Qué le quise decir con eso? Dígame con sus palabras lo que usted entendió
- ¿Qué significa biobanco?
- ¿Cuáles son sus preocupaciones?

Señor (a) XXX., las muestras de ADN y la información se utilizarán para la investigación, *y dicho uso podría dar lugar a invenciones y descubrimientos que podrían convertirse en la base de nuevos productos comerciales, pruebas de diagnóstico o agentes terapéuticos.*

Voy a leer nuevamente: las muestras de ADN y la información se utilizarán para la investigación, *y dicho uso podría dar lugar a invenciones…*

- ¿Qué le quise decir con eso? Dígame con sus palabras lo que usted entendió
- Usted mencionó a XX, cuénteme más sobre eso.
- Cuando le dije “nuevos productos comerciales, pruebas de diagnóstico o agentes terapéuticos” ¿qué cree que le quise decir?
- ¿Qué siente al pensar que usted (o su hijo) participaran en este estudio?

Vamos con otro párrafo:

Señor (a) XXX., con respecto a este estudio de zika, aunque ni usted ni su hijo ni los investigadores se beneficiarán directa o comercialmente de esta parte de la investigación, las generaciones futuras podrán beneficiarse de los conocimientos que se obtengan.

Quiero leer nuevamente la siguiente frase, para que usted me diga qué entiende de ella:

Aunque ni usted, ni su hijo, ni los investigadores se beneficiarán directa o comercialmente de esta parte de la investigación, las generaciones futuras podrán beneficiarse de los conocimientos que se obtengan.

- ¿Qué le quise decir con eso? Dígame con sus palabras lo que usted entendió
- Usted mencionó a XX, cuénteme más sobre eso.
- ¿Qué significa beneficiarse directamente?
- ¿Qué significa beneficiarse comercialmente?

Vamos con otro párrafo:

Señor (a) XXX., *las muestras de ADN y la información clínica suya (o de su hijo) se pondrán a* disposición del Instituto Arco iris y de otros investigadores sólo si usted está de acuerdo con este procedimiento.

Voy a leer nuevamente: *las muestras de ADN y la información clínica de su hijo se pondrán a….*

- ¿Qué le quise decir con eso? Dígame con sus palabras lo que usted entendió
- Usted mencionó a XX, cuénteme más sobre eso.
- Cuando le digo que *“solo si usted está de acuerdo con este procedimiento”* ¿a cuál procedimiento cree que me refiero?

Ya estamos terminando, vamos con este párrafo:

Señor (a) XXX., Señor (a) XXX., *las muestras de ADN y la información clínica suya (o de su hijo)* recibirán un número de identificación único. La muestras, no tendrán ninguna información que pueda identificarle a usted o a su hijo (como el nombre o la dirección). El Instituto Arco Iris y otros investigadores no tendrán acceso a la clave que vincula la identidad de su hijo con la información y las muestras.

Voy a leer nuevamente: *las muestras de ADN y la información clínica de su hijo recibirán un número…*

- ¿Qué le quise decir con eso? Dígame con sus palabras lo que usted entendió
- Usted mencionó a XX, cuénteme más sobre eso.
- Yo le acabo de leer esta frase: las muestras, no tendrán ninguna información que pueda identificarle a usted o a su hijo (como el nombre o la dirección). ¿Qué le quise decir con eso?
- También le leí lo siguiente: el Instituto Arco Iris y otros investigadores no tendrán acceso a la clave que vincula la identidad de su hijo con la información y las muestras. En otras palabras, el nombre de su hijo se mantendrá en secreto.

¿Qué le quise decir con eso?

- ¿Qué piensa usted sobre la forma en que manejaremos las muestras de sangre y la información genética de su hijo?
- ¿Qué cree que sucederá con la información que fue recolectada de su hijo/usted?
- ¿Cuáles son sus preocupaciones?

Señor (a) XXX., *si más adelante decide que no quiere que las muestras de ADN de su hijo se utilicen para* futuras investigaciones genéticas, comuníquese con nosotros y haremos todo lo posible para evitar su uso en cualquier otro estudio.

Voy a leer nuevamente: *si más adelante decide que no quiere que las muestras de ADN de su hijo se utilicen para…*

- ¿Qué le quise decir con eso? Dígame con sus palabras lo que usted entendió
- Usted mencionó a XX, cuénteme más sobre eso.
- Yo leí lo siguiente: si usted decide que no quiere que las muestras de ADN de su hijo se utilicen para futuras investigaciones genéticas, comuníquese con nosotros y haremos todo lo posible para evitar su uso en cualquier otro estudio.
- ¿De qué manera se comunicaría con nosotros? ¿Y qué me diría?

Le voy a leer otro párrafo:

Señor (a) XXX., la participación en futuros estudios genéticos *no es obligatoria y no habrá consecuencias* si usted no quiere que las muestras o los datos de su hijo se utilicen en futuros estudios genéticos.

Voy a leer nuevamente: la participación en futuros estudios genéticos *no es obligatoria y no habrá consecuencias…*

- Cuando le digo que *“no habrá consecuencias” ¿*a qué cree que me refiero?
- ¿Qué piensa usted sobre la forma en que manejaremos las muestras de sangre y la información genética de su hijo?
- ¿Cuáles son sus preocupaciones?

Muy bien señor (a) XXX., ahora usted es un experto en manejo de muestras y datos. Le voy a leer todo el párrafo completo para que usted al final elija una opción. ¿Listo?

Las muestras de ADN y la información clínica de su hijo se pondrán a disposición del Instituto Arco iris y de otros investigadores sólo si usted está de acuerdo con este procedimiento. Las muestras y la información de su hijo recibirán un número de identificación único. La muestras, no tendrán ninguna información que pueda identificarle a usted o a su hijo (como el nombre o la dirección). El Instituto Arco Iris y otros investigadores no tendrán acceso a la clave que vincula la identidad de su hijo con la información y las muestras. En otras palabras, el nombre de su hijo se mantendrá en secreto. Si más adelante decide que no quiere que las muestras de ADN de su hijo se utilicen para futuras investigaciones genéticas, comuníquese con nosotros y haremos todo lo posible para evitar su uso en cualquier otro estudio. La participación en futuros estudios genéticos no es obligatoria y no habrá consecuencias si usted no quiere que las muestras o los datos de su hijo se utilicen en futuros estudios genéticos.

Por favor, marque la casilla de abajo que indica su voluntad de proporcionar las muestras de ADN y la información clínica de su hijo para futuras investigaciones genéticas.

1. Estoy de acuerdo en permitir que la información clínica, las muestras y el ADN contenido en ellas sean usados para estudios genéticos futuros y compartidos con otros colaboradores.
2. NO estoy de acuerdo en permitir que la información clínica, las muestras y el ADN contenido en ellas sean usados para estudios genéticos futuros y compartidos con otros colaboradores.

*Le vuelvo a leer… ¿desea que lea todo el párrafo o únicamente las preguntas? (lea el párrafo o las preguntas)*

*Bien, antes de conocer su respuesta, me gustaría saber lo siguiente:*

- ¿Qué le quise decir con cada pregunta? ¿Lo recuerda? ¿sobre qué tenía que elegir? ¿Cuáles eran las opciones?

*Explicar las opciones si no fueron claras para el participante*…

Ahora por favor dígame con sus palabras, cuales son las opciones entre las que va a elegir

Muy bien. Ahora, dígame, de esas dos opciones, ¿cuál elegiría usted?

¿Qué le hizo escoger esta opción?

- ¿Cómo cree que esta su nivel de comprensión en relación con el manejo de información genética de su hijo?
- Porque cree que se mantiene / o ha mejorado / o ha empeorado

Excelente señor (a) XXX., hizo un gran trabajo y sus respuestas nos ayudan a comprender, si esta información relacionada con muestras biológicas y uso futuro es clara para usted como participante o no lo es.

¿Qué más le gustaría saber acerca de cómo el estudio manejaría la información o las muestras, como sangre, recolectada de su hijo?

¿Qué más le gustaría saber acerca de cómo el estudio compartirá la información genética de su hijo?

¿Qué le diría al equipo de investigación sobre el intercambio de datos o muestras de los participantes para otros estudios que tienen como objetivo beneficiar la salud pública?

¿Qué piensa usted de que las muestras sean compartidas con organizaciones vinculadas a la salud pública o a la industria farmacéutica?

¿Qué piensa usted acerca de que su muestra y la información sea enviada a otras instituciones de investigación  fuera del país? (aquí se puede contextualizar acerca del procedimiento).

¿Qué le diría a un participante a quien están invitando a participar en una investigación y le pedirán permiso para compartir datos o muestras en estudios que beneficien la salud pública?

**Supplementary Text S2. Interview guide that evaluates understanding of language from the Spanish translation of the University of California at Berkeley Spanish-language template informed consent for biomedical studies updated with language from explanations from research participants, in track changes**

# Entrevista cognitiva modificada con lenguaje de la comunidad

***Introducción a la entrevista***

Señor (a) XXX., vamos a hablar de algunos temas que generalmente se mencionan, cuando se contacta a las personas para que participen en estudios de investigación que incluyen un consentimiento amplio. Como usted ha participado en ese tipo de estudios es muy probable que algunas frases ya le sean familiares. Si no, no hay problema, poco a poco las vamos a recordar.

Señor (a) XXX., durante esta entrevista, vamos a hacer un ejercicio que se llama “parafraseo para la comprensión”. Para hacerlo vamos “a hacer de cuenta” que lo / la estoy invitando a participar de un estudio y al final le pediré que me diga “con sus palabras” qué entiende por algunas frases o palabras relacionadas con el uso de muestras y datos para estudios futuros.

Recuerde señor (a) XXX., este ejercicio lo vamos a hacer sobre un estudio que no es real, pero que se lo voy a narrar como si lo fuera.

¿Está listo / a? Recuerde, ahora yo soy un investigador y usted es un /a posible candidato/ a para participar en un estudio. ¿Empezamos con la primera parte?

*A. Introducir el lenguaje del consentimiento informado amplio*

Señor (a) XXX., muy buena tarde. Lo contacto para invitarlo a participar en el estudio “Grado de afectación del ZIKA en la población infantil y adulta de Colombia”. *(Para contextualizar al participante, se le hará un corto resumen sobre ¿Qué es el zika? ¿Quiénes pueden participar? ¿Objetivo del estudio? ¿Procedimientos a realizar durante el estudio? ¿Riesgos y beneficios?)*

Señor (a) XXX., ¿tiene alguna pregunta en relación con el estudio? Muy bien, una vez aclaradas sus inquietudes, en adelante, le voy a solicitar que nos permita compartir las muestras de sangre que tomaremos en el estudio.

Excelente trabajo Señor (a) XXX., ahora vamos a iniciar la segunda parte que está relacionada con el uso de las muestras en estudios futuros. Recuerde que estamos “haciendo de cuenta” que lo estoy invitando a participar en el estudio “Grado de afectación del zika en la población infantil y adulta de Colombia”. Ya le dije de qué se trata el estudio; ahora, vamos a revisar algunos temas relacionados con el uso futuro de muestras. ¿Continuamos?

Señor (a) XXX., quiero comentarle que para este estudio del zika, “*tenemos previsto…”* **Uso futuro**

*Tenemos previsto* conservar y compartir la información y las muestras recogidas de su hijo en caso de que nosotros u otros investigadores queramos utilizarlas posteriormente para otros estudios.

Voy a repetir XXX: *tenemos previsto*…

*Indagar*

∙ ¿Qué le quise decir con eso? dígame con sus palabras lo que le acabo de decir. ∙ ¿En qué pensó usted cuando escuchó esto?

∙ Usted mencionó XXXX, cuénteme más sobre eso.

∙ Cuando digo que vamos a conservar y compartir “muestras,” ¿qué significa para usted? ¿qué entiende por “muestras biológicas”?

∙ ¿Qué cree que pasará con la sangre de su hijo que fue recogida?

Excelente, vamos con otra frase. El mismo ejercicio pero con otra frase:

Señor (a) XXX., quiero comentarle que para este estudio del zika, *la información de identificación*, como el nombre, la dirección y la fecha de nacimiento de su hijo, se eliminará del registro de su hijo o del registro asociado a su muestra antes de que compartamos sus datos o su muestra con cualquier otro grupo de investigación.

Le vuelvo a leer… *la información de identificación*…

∙ ¿Qué le quise decir con eso? dígame con sus palabras lo que le acabo de decir. ∙ ¿Qué cree que significa, que la información de identificación de su hijo se eliminara del registro asociado a su muestra?

∙ Usted mencionó a XX, cuénteme más sobre eso.

Excelente, vamos con otra frase. El mismo ejercicio pero con otra frase:

Señor (a) XXX., quiero comentarle que en este estudio del zika, *después de eliminar cualquier información de identificación*, la información o las muestras recogidas de su hijo podrían ser utilizadas por nuestro equipo o por otros investigadores para futuros estudios, sin el consentimiento informado adicional de su hijo o de usted mismo.

Le vuelvo a leer… *después de eliminar cualquier información de identificación…*

∙ ¿Qué le quise decir con eso? dígame con sus palabras lo que le acabo de decir ∙ ¿Qué cree que significa, que nuestro equipo podría usar la información o la sangre recolectado de su hijo para otros estudios o compartirlos con otros equipos de investigación?

∙ Y ¿Cómo se siente usted cuando escucha eso?

∙ Usted dice XXXXX…. explícame un poco más acerca de esto.

Muy bien señor (a) XXX., ahora, si le digo que pensamos compartir las respuestas o las muestras, como la sangre, con equipos de investigación de otros países para, por ejemplo, desarrollar nuevas vacunas para el zika…

∙ ¿Qué le quise decir con eso? dígame con sus palabras lo que le acabo de decir ∙ Y ¿Cómo se siente usted cuando escucha eso?

∙ Usted dice XXXXX…. explícame un poco más acerca de esto.

Excelente, vamos con otra frase:

Señor (a) XXX., quiero comentarle que *cualquier estudio que utilice los datos o las muestras* de su hijo recolectadas en este estudio del zika, tendrá que proporcionar información sobre cómo se mantendrá la confidencialidad de los datos o las muestras de su hijo

Le vuelvo a leer… *cualquier estudio que utilice los datos o las muestras…*

∙ ¿Qué le quise decir con eso? dígame con sus palabras lo que le acabo de decir ∙ ¿Qué significa mantener la confidencialidad?

∙ Y ¿Cómo se siente usted al permitir que su hijo participe en ese estudio? ∙ Usted dice XXXXX…. explícame un poco más acerca de esto.

Señor (a) XXX., quiero comentarle que *cualquier estudio que utilice los datos o las muestras* de su hijo recolectadas en este estudio del zika, tendrá que proporcionar información sobre cómo se mantendrá la confidencialidad de los datos o las muestras de su hijo y cómo la futura investigación podría beneficiar a su comunidad o a la salud pública en general.

Le vuelvo a leer… *cualquier estudio que utilice los datos o las muestras…*

¿Qué le quise decir con eso? dígame con sus palabras lo que le acabo de decir

¿Cómo entiende usted los beneficios que puede recibir la comunidad? ¿Qué significa beneficios para la salud pública?

¿Cómo se siente de participar / usted o su hijo/ en este estudio?

Vamos nuevamente con otras frases:

**Cláusula Moore (información para el investigador, no para el participante)**

Señor (a) XXX., quiero comentarle que *las muestras recogidas de su hijo para* este estudio del zika, y/o la información obtenida de esas muestras, pueden utilizarse en esta investigación o en otras investigaciones, y compartirse con otras organizaciones.

Le vuelvo a leer… *las muestras recogidas de su hijo para el…*

∙ ¿Qué le quise decir con eso? dígame con sus palabras lo que le acabo de decir ∙ ¿Qué significa, que la información obtenida de esas muestras, compartirse con otras organizaciones?

∙ Y ¿Cómo se siente usted al permitir que su hijo participe en ese estudio? ∙ Usted dice XXXXX…. explícame un poco más acerca de esto.

Señor (a) XXX., quiero comentarle que las muestras recogidas de su hijo (o suyas) para este estudio del zika, y/o la información obtenida de esas muestras, pueden compartirse con otras organizaciones y usted no participará de ningún valor comercial o beneficio derivado del uso de las muestras de su hijo y/o de la información obtenida de ellos.

Le vuelvo a leer… *las muestras recogidas de su hijo para el…*

∙ ¿Qué le quise decir con eso? dígame con sus palabras lo que le acabo de decir ∙ ¿Qué significa, que usted no participará de ningún valor comercial o beneficio derivado del uso de las muestras de su hijo?

∙ Y ¿Cómo se siente usted al permitir que su hijo participe en ese estudio? ∙ Usted dice XXXXX…. explícame un poco más acerca de esto.

Señor (a) XXX., quiero comentarle que este estudio del zika, incluirá la secuenciación del ADN o ARN del genoma completo.

Le vuelvo a leer… *quiero comentarle que este estudio del zika…*

∙ ¿Qué le quise decir con eso? dígame con sus palabras lo que le acabo de decir ∙ ¿Qué significa, incluir la secuenciación del ADN o ARN del genoma?

∙ Y ¿Cómo se siente usted al permitir que su hijo participe en ese estudio? ∙ Usted dice XXXXX…. explícame un poco más acerca de esto.

∙ Para usted, ¿qué quiere decir genoma?

Señor (a) XXX., quiero comentarle que *los resultados de* este estudio del zika que sean clínicamente relevantes, incluyendo los resultados individuales de la investigación, no serán revelados a los sujetos participantes

Le vuelvo a leer… *los resultados de la investigación…*

∙ ¿Qué le quise decir con eso? dígame con sus palabras lo que le acabo de decir ∙ ¿Qué entiende por resultados clínicamente relevantes? Por favor, dígame lo mismo con sus propias palabras

∙ ¿Qué entiende por resultados individuales de la investigación?

∙ Usted dice XXXXX…. explícame un poco más acerca de esto.

Excelente señora XXX. Ahora voy a leer nuevamente todo el texto pero, esta vez, al final usted va a tomar una decisión con base en la información. ¿Listo?

Las muestras recogidas de su hijo para este estudio y/o la información obtenida de esas muestras, pueden utilizarse en esta investigación o en otras investigaciones, y compartirse con otras organizaciones. Usted no participará de ningún valor comercial o beneficio derivado del uso de las muestras de su hijo y/o de la información obtenida de ellos. La investigación incluirá la secuenciación del ADN o ARN del genoma completo. Los resultados de la investigación clínicamente relevantes, incluyendo los resultados individuales de la investigación, no serán revelados a los sujetos participantes. Calculamos que nuestro estudio tardará 5 años o más en completarse.

Por favor, elija una de las siguientes opciones:

1. Usted permite que las muestras de su hijo se utilicen para este estudio y se almacenen para futuros estudios o

2. Usted permite que las muestras de su hijo se utilicen para este estudio PERO solicita que se destruyan después de la finalización de este estudio y no se utilicen en futuros estudios

*Le vuelvo a leer… ¿desea que lea todo el párrafo o únicamente las preguntas? Bien, antes de conocer su respuesta, me gustaría saber lo siguiente:*

∙ ¿Qué le quise decir con cada pregunta? ¿Lo recuerda? ¿sobre qué tenía que elegir? ¿Cuáles eran las opciones?

*Explicar las opciones si no fueron claras para el participante*…

∙ Ahora, si usted estuviera participando nuevamente en ese estudio, y tuviera que elegir entre esas dos opciones ¿Cual escogería usted?

∙ ¿Por qué? Cuéntame más acerca de esto.

Y si usted dijo que NO DA PERMISO, ¿hasta cuándo piensa que se guardaran las muestras?

Muy bien señor XXX continuemos con el ejercicio. Lo está haciendo muy bien. Es genial hacer este tipo de trabajo con personas como usted.

**Datos genéticos (información para el investigador, no para el participante)** Vamos con esta frase:

Señor (a) XXX., quiero comentarle que para este estudio del zika *nos gustaría contar con un permiso* adicional para almacenar las muestras de sangre y el ADN de su hijo para futuros estudios genéticos. Los estudios genéticos examinan la información genética que se transmite de padres a hijos.

Le vuelvo a leer… quiero comentarle que para este estudio del zika *nos gustaría contar con un permiso…*

∙ ¿Qué le quise decir con eso? Dígame con sus palabras lo que usted entendió ∙ Usted mencionó XX, cuénteme más sobre eso.

∙ Usted menciono XXXX cuénteme más acerca de esto.

Señor (a) XXX., quiero comentarle que para este estudio del zika, en concreto, buscaremos genes que puedan hacer que las personas tengan más o menos probabilidades de desarrollar enfermedades graves causadas por infecciones de virus como el zika, Chikunguña y otros genes relacionados con el estado de salud.

∙ ¿Recuerda que en algunas preguntas anteriores hablamos sobre datos genéticos? Entonces quiero saber, como está ahora su comprensión sobre ese tema, sobre datos genéticos ∙ ¿Qué significa que vamos a buscar genes?

Señor (a) XXX., *se espera que esta investigación* sobre el zika *dure muchos años.* Si usted decide no darnos su permiso, puede seguir participando en el estudio y esto no afectará a la atención médica que recibe su hijo.

Voy a leer nuevamente: *se espera que esta investigación* sobre el zika *dure muchos años…*

∙ ¿Qué le quise decir con eso? Dígame con sus palabras lo que usted entendió ∙ Usted mencionó a XX, cuénteme más sobre eso.

∙ ¿Cuántos años cree que durará el estudio?

∙ Para usted qué significa confidencialidad de la información.

Señor (a) XXX., *las muestras de ADN y la información clínica se enviarán, sin el nombre* de su hijo, a uno de nuestros socios de investigación y podrán enviarse a otros grupos de investigación que colaboren con nuestro laboratorio en el futuro.

Voy a leer nuevamente: *las muestras de ADN y la información clínica se enviarán, sin el nombre…*

∙ ¿Qué le quise decir con eso? Dígame con sus palabras lo que usted entendió ∙ Usted mencionó a XX, cuénteme más sobre eso.

∙ ¿Qué significa para usted, muestras de ADN?

∙ ¿Qué piensa usted sobre la forma en que manejaremos las muestras de sangre y la información genética de su hijo?

∙ ¿Cuáles son sus preocupaciones?

Señor (a) XXX., cualquier muestra adicional *será almacenada en un biobanco* de Colombia.

∙ ¿Qué le quise decir con eso? Dígame con sus palabras lo que usted entendió ∙ ¿Qué significa biobanco?

∙ ¿Cuáles son sus preocupaciones?

Señor (a) XXX., las muestras de ADN y la información se utilizarán para la investigación, *y dicho uso podría dar lugar a invenciones y descubrimientos que podrían convertirse en la base de nuevos productos comerciales, pruebas de diagnóstico o agentes terapéuticos.*

Voy a leer nuevamente: las muestras de ADN y la información se utilizarán para la investigación, *y dicho uso podría dar lugar a invenciones…*

∙ ¿Qué le quise decir con eso? Dígame con sus palabras lo que usted entendió ∙ Usted mencionó a XX, cuénteme más sobre eso.

∙ Cuando le dije “nuevos productos comerciales, pruebas de diagnóstico o agentes terapéuticos” ¿qué cree que le quise decir?

∙ ¿Qué siente al pensar que usted (o su hijo) participaran en este estudio? Vamos con otro párrafo:

Señor (a) XXX., con respecto a este estudio de zika, aunque ni usted ni su hijo ni los investigadores se beneficiarán directa o comercialmente de esta parte de la investigación, las generaciones futuras podrán beneficiarse de los conocimientos que se obtengan.

Quiero leer nuevamente la siguiente frase, para que usted me diga qué entiende de ella:

Aunque ni usted, ni su hijo, ni los investigadores se beneficiarán directa o comercialmente de esta parte de la investigación, las generaciones futuras podrán beneficiarse de los conocimientos que se obtengan.

∙ ¿Qué le quise decir con eso? Dígame con sus palabras lo que usted entendió ∙ Usted mencionó a XX, cuénteme más sobre eso.

∙ ¿Qué significa beneficiarse directamente?

∙ ¿Qué significa beneficiarse comercialmente?

Vamos con otro párrafo:

Señor (a) XXX., *las muestras de ADN y la información clínica suya (o de su hijo) se pondrán a* disposición del Instituto Arco iris y de otros investigadores sólo si usted está de acuerdo con este procedimiento.

Voy a leer nuevamente: *las muestras de ADN y la información clínica de su hijo se pondrán a….*

∙ ¿Qué le quise decir con eso? Dígame con sus palabras lo que usted entendió ∙ Usted mencionó a XX, cuénteme más sobre eso.

∙ Cuando le digo que *“solo si usted está de acuerdo con este procedimiento”* ¿a cuál procedimiento cree que me refiero?

Ya estamos terminando, vamos con este párrafo:

Señor (a) XXX., Señor (a) XXX., *las muestras de ADN y la información clínica suya (o de su hijo)* recibirán un número de identificación único. La muestras, no tendrán ninguna información que pueda identificarle a usted o a su hijo (como el nombre o la dirección). El Instituto Arco Iris y otros investigadores no tendrán acceso a la clave que vincula la identidad de su hijo con la información y las muestras.

Voy a leer nuevamente: *las muestras de ADN y la información clínica de su hijo recibirán un número…*

∙ ¿Qué le quise decir con eso? Dígame con sus palabras lo que usted entendió ∙ Usted mencionó a XX, cuénteme más sobre eso.

∙ Yo le acabo de leer esta frase: las muestras, no tendrán ninguna información que pueda identificarle a usted o a su hijo (como el nombre o la dirección). ¿Qué le quise decir con eso? ∙ También le leí lo siguiente: el Instituto Arco Iris y otros investigadores no tendrán acceso a la clave que vincula la identidad de su hijo con la información y las muestras. En otras palabras, el nombre de su hijo se mantendrá en secreto.

¿Qué le quise decir con eso?

∙ ¿Qué piensa usted sobre la forma en que manejaremos las muestras de sangre y la información genética de su hijo?

∙ ¿Qué cree que sucederá con la información que fue recolectada de su hijo/usted? ∙ ¿Cuáles son sus preocupaciones?

Señor (a) XXX., *si más adelante decide que no quiere que las muestras de ADN de su hijo se utilicen para* futuras investigaciones genéticas, comuníquese con nosotros y haremos todo lo posible para evitar su uso en cualquier otro estudio.

Voy a leer nuevamente: *si más adelante decide que no quiere que las muestras de ADN de su hijo se utilicen para…*

∙ ¿Qué le quise decir con eso? Dígame con sus palabras lo que usted entendió ∙ Usted mencionó a XX, cuénteme más sobre eso.

∙ Yo leí lo siguiente: si usted decide que no quiere que las muestras de ADN de su hijo se utilicen para futuras investigaciones genéticas, comuníquese con nosotros y haremos todo lo posible para evitar su uso en cualquier otro estudio.

∙ ¿De qué manera se comunicaría con nosotros? ¿Y qué me diría?

Le voy a leer otro párrafo:

Señor (a) XXX., la participación en futuros estudios genéticos *no es obligatoria y no habrá consecuencias* si usted no quiere que las muestras o los datos de su hijo se utilicen en futuros estudios genéticos.

Voy a leer nuevamente: la participación en futuros estudios genéticos *no es obligatoria y no habrá consecuencias…*

∙ Cuando le digo que *“no habrá consecuencias” ¿*a qué cree que me refiero? ∙ ¿Qué piensa usted sobre la forma en que manejaremos las muestras de sangre y la información genética de su hijo?

∙ ¿Cuáles son sus preocupaciones?

Muy bien señor (a) XXX., ahora usted es un experto en manejo de muestras y datos. Le voy a leer todo el párrafo completo para que usted al final elija una opción. ¿Listo?

Las muestras de ADN y la información clínica de su hijo se pondrán a disposición del Instituto Arco iris y de otros investigadores sólo si usted está de acuerdo con este procedimiento. Las muestras y la información de su hijo recibirán un número de identificación único. La muestras, no tendrán ninguna información que pueda identificarle a usted o a su hijo (como el nombre o la dirección). El Instituto Arco Iris y otros investigadores no tendrán acceso a la clave que vincula la identidad de su hijo con la información y las muestras. En otras palabras, el nombre de su hijo se mantendrá en secreto. Si más adelante decide que no quiere que las muestras de ADN de su hijo se utilicen para futuras investigaciones genéticas, comuníquese con nosotros y haremos todo lo posible para evitar su uso en cualquier otro estudio. La participación en futuros estudios genéticos no es obligatoria y no habrá consecuencias si usted no quiere que las muestras o los datos de su hijo se utilicen en futuros estudios genéticos.

Por favor, marque la casilla de abajo que indica su voluntad de proporcionar las muestras de ADN y la información clínica de su hijo para futuras investigaciones genéticas.

1. Estoy de acuerdo en permitir que la información clínica, las muestras y el ADN contenido en ellas sean usados para estudios genéticos futuros y compartidos con otros colaboradores.

2. NO estoy de acuerdo en permitir que la información clínica, las muestras y el ADN contenido en ellas sean usados para estudios genéticos futuros y compartidos con otros colaboradores.

*Le vuelvo a leer… ¿desea que lea todo el párrafo o únicamente las preguntas? (lea el párrafo o las preguntas)*

*Bien, antes de conocer su respuesta, me gustaría saber lo siguiente:*

∙ ¿Qué le quise decir con cada pregunta? ¿Lo recuerda? ¿sobre qué tenía que elegir? ¿Cuáles eran las opciones?

*Explicar las opciones si no fueron claras para el participante*…

Ahora por favor dígame con sus palabras, cuales son las opciones entre las que va a elegir Muy bien. Ahora, dígame, de esas dos opciones, ¿cuál elegiría usted?

¿Qué le hizo escoger esta opción?

∙ ¿Cómo cree que esta su nivel de comprensión en relación con el manejo de información genética de su hijo?

∙ Porque cree que se mantiene / o ha mejorado / o ha empeorado

Excelente señor (a) XXX., hizo un gran trabajo y sus respuestas nos ayudan a comprender, si esta información relacionada con muestras biológicas y uso futuro es clara para usted como participante o no lo es.

¿Qué más le gustaría saber acerca de cómo el estudio manejaría la información o las muestras, como sangre, recolectada de su hijo?

¿Qué más le gustaría saber acerca de cómo el estudio compartirá la información genética de su hijo?

¿Qué le diría al equipo de investigación sobre el intercambio de datos o muestras de los participantes para otros estudios que tienen como objetivo beneficiar la salud pública?

¿Qué piensa usted de que las muestras sean compartidas con organizaciones vinculadas a la salud pública o a la industria farmacéutica?

¿Qué piensa usted acerca de que su muestra y la información sea enviada a otras instituciones de investigación fuera del país? (aquí se puede contextualizar acerca del procedimiento).

¿Qué le diría a un participante a quien están invitando a participar en una investigación y le pedirán permiso para compartir datos o muestras en estudios que beneficien la salud pública?
