# Supplementary material for: "How about me giving blood for the COVID vaccine and not being able to get vaccinated?" A cognitive interview study on understanding of and agreement with broad consent for future use of data and samples in Colombia and Nicaragua
Source: PLOS Glob Public Health. 2023 May 17;3(5):e0001253. doi: 10.1371/journal.pgph.0001253 (PMC10191364; doi:10.1371/journal.pgph.0001253)
Supplement: S3 Text — (DOCX) [file pgph.0001253.s007.docx]

Inclusivity in global research

PLOS’ policy on inclusivity in global research aims to improve transparency in the reporting of research performed outside of researchers’ own country or community and ensures that PLOS publications reporting global research adhere to high standards for research ethics and authorship. Authors of relevant research articles may be asked to complete the questionnaire below, which outlines ethical, cultural, and scientific considerations specific to inclusivity in global research. This questionnaire may be requested when researchers have travelled to a different country to conduct research, if research uses samples collected in another country, research with Indigenous populations or their lands, or if research is on cultural artefacts. Researchers travelling to another country solely to use laboratory equipment will not normally be required to complete the questionnaire. However, the questionnaire can be requested at the journal’s discretion for any submission – if you have been requested to complete this questionnaire by the PLOS journal you submitted to, please do so.

Please complete the questionnaire below and include this as a Supporting Information file with your manuscript. Note that if your paper is accepted for publication, this checklist will be published with your article in the supporting information files. Please ensure that you reference the checklist in the main body of your manuscript. We suggest adding a subsection ‘Inclusivity in global research’ to your Methods section and adding the following sentence: “Additional information regarding the ethical, cultural, and scientific considerations specific to inclusivity in global research is included in the Supporting Information (SX Checklist)”

The questions have been designed to be applicable to a wide range of study types, and there are subsections for both human subjects research and non-human subjects research. If any of the questions are not relevant to your research please mark them as “N/A” as appropriate.

**Ethical considerations, permits and authorship**

*This section is applicable to all research types.*

Provide details as to who granted permissions and/or consent for the study to take place in the Methods section of your manuscript. This should include the names of **all** ethics boards, governmental organizations, community leaders or other bodies that provided approval for the study. If individuals provided approval refer to these people by their role or title but do not list their name(s).

Reported on page 13. The project was reviewed and approved by the Comité de Ética en Investigación Biomédica CDI (CEIB-2719-2021; Bucaramanga, Colombia), the Instituto de Ciencias Sostenibles (Managua, Nicaragua), and the Universitätsklinikum Heidelberg (S-312/2021; Heidelberg, Germany) Ethics Review Committees.

If there were any deviations from the study protocol after approval was obtained please provide details of these changes in the Methods section of your manuscript.
Did this study involve local collaborators that are residents of the country where the research was conducted or members of the community studied? If you do not have any authors from said communities, please provide an explanation for this below.

The study was designed, managed, and reported by local collaborators that are residents of Colombia and Nicaragua, where the data collection was conducted.

There were no deviations from the protocol following approval.

Everyone listed as an author should meet PLOS’ criteria for authorship and all individuals who meet these criteria should be included in the author byline, rather than the acknowledgements. Authorship criteria is based on the International Committee of Medical Journal Editors (ICMJE) Uniform Requirements for Manuscripts Submitted to Biomedical Journals - for further information please see here: <https://journals.plos.org/plosone/s/authorship>.

**Human subjects research (e.g. health research, medical research, cross-cultural psychology)**

Did you obtain written informed consent from a representative of the local community or region before the research took place? How did you establish who speaks for the community? Details of written informed consent obtained from study participants should be reported separately in the Methods section of your manuscript.

We did not receive written informed consent from a representative of the local community before the research took place. We report details of the informed consent process for research participants in the mansusript, page 8.

How did members of the local community provide input on the aims of the research investigation, its methodology, and its anticipated outcome(s)?

Members of the source cohort research team were engaged in protocol development and the execution and analysis of the data. Research team members are from the same community as cohort participants (Nicaragua) or have lived and worked in that community (Colombia). We conducted 3 pilot interviews in Nicaragua and four in Colombia. In these pilot interviews, we reviewed the informed consent for the study, the cognitive interview practice exercise and guide, and the semi-structured interview guide. The study informed consent and the structure of the study interview were changed following these pilot interviews to make thte material easier to get through. See page 10 for details.

Participants in Colombia were interviewed by a Colombian nurse and bioethicist and in Nicaragua by a Nicaraguan economist and expert in community-based participatory research. Both have been involved in the long-term cohorts are involved in data and sample sharing-related research, and are experienced in conducting and analyzing qualitative research, including cognitive interviewing. The team members in Nicaragua and Colombia are familiar with the local context having worked in the communities where the research was conducted or being from those communiities. They are also knowledgeable about the language of the participants and the meaning of words in the context in which they are used.

When engaging with the local community, how did you ensure that the informed consent documents and other materials could be understood by local stakeholders

The research team developed an informed consent form that explained the purpose of the study, the reasons for inviting the participant, the study procedures, the risks and benefits, how confidentiality of information would be maintained, and the participant's rights as a participant. Comprehension of the information was ensured by asking the participant to state in his or her own words the information provided in the informed consent and then to confirm his or her agreement to participate in the study.

Will the findings of the research be made available in an understandable format to stakeholders in the community where the study was conducted (e.g. via a presentation, summary report, copies of publications, etc.)? Please provide details of how this will be achieved.

The results of the study will be shared in an open access, peer-reviewed scientific journal and at related scientific and health research ethics events.

**Non-human subjects research using specimens/ animals collected as part of the study, or those housed in archival collections. Examples include archaeology, paleontology, botany and zoology.**

Did the permission you obtained from a local authority to perform the study include an agreement on access to outputs and benefit sharing? This may include procedures to enable fair distribution of the benefits and resources arising from the research performed. Please include any details of Prior Informed Consent and Benefit Sharing Agreements obtained. These may be required by field-specific regulations, for example the Convention on Biological Diversity (CBD) and the associated Nagoya Protocol.

N/A

If the material used in your study was imported, please A) provide the year it was imported and B) indicate whether permits were obtained to import/export the materials used, C) provide details of any permits obtained. If this information is not available, please indicate this.

N/A

If you used archival specimens, please state how the material used in your study was acquired by the institute it is held in and provide details of any permits obtained for the original excavations/ sample collection. If this information is not available, please indicate this.

N/A

How was the potential cultural significance of the materials collected in your study to local communities considered in your research design? Were Indigenous peoples and/or local researchers and institutions involved with archaeological excavations / collection of specimens? If so, please provide a description of their involvement.

N/A

If your manuscript includes photographs of human remains please indicate whether authors obtained permission from descendants or affiliated cultural communities to do so.

N/A
